# The mitochondrial protease PARL is required for spermatogenesis

**DOI:** 10.1038/s42003-023-05703-3

**Published:** 2024-01-05

**Authors:** Sarah Schumacher, Laura Klose, Jessica Lambertz, Dieter Lütjohann, Ronald Biemann, Stefanie Kuerten, Lars Fester

**Affiliations:** 1 Institute of Neuroanatomy, Medical Faculty, University of Bonn, 53115 Bonn, Germany; 2 Institute of Clinical Chemistry and Clinical Pharmacology, University Hospital Bonn, 53127 Bonn, Germany; 3 Institute of Laboratory Medicine, Clinical Chemistry and Molecular Diagnostics, University of Leipzig, 04103 Leipzig, Germany

**Keywords:** Spermatogenesis, Energy metabolism, Steroid hormones

## Abstract

Mitochondrial function plays an important role in the maintenance of male fertility. However, the mechanisms underlying mitochondrial defect-related infertility remain mostly unclear. Here we show that a deficiency of PARL (*Parl*^−/−^), a mitochondrial protease, causes complete arrest of spermatogenesis during meiosis I. PARL deficiency led to severe downregulation of proteins of respiratory chain complex IV in testes that did not occur in other tested organs, causing a deficit in complex IV activity and ATP production. Furthermore, *Parl*^−/−^ testes showed an almost complete loss of HSD17B3, a protein of the sER responsible for the last step in testosterone synthesis. While testosterone production appeared to be restored by overexpression of HSD17B12, loss of the canonical testosterone synthesis led to an upregulation of luteinizing hormone (LH) and of LH-regulated responses. These results suggest an important impact of the downstream regulation of mitochondrial defects that manifest in a cell-type-specific manner and extend beyond mitochondria.

## Abstract (web summary)

The mitochondrial protease PARL is essential for male fertility in mice, and knockout of PARL leads to an arrest in spermatogenesis due to downregulation of respiratory chain complex IV proteins in the testes and a deficit in ATP production there.

## Introduction

Male infertility is an increasing clinical problem that affects up to 7% of the male population^1^. Non-obstructive azoospermia, the most severe form of infertility, manifests in complete arrest of spermatogenesis, leaving patients no chance of producing offspring without severe medical intervention^2^. The impact of mitochondrial disease on male infertility has been discussed for many years, and evidence is increasing that correct mitochondrial function is essential at several levels for male fertility, including sperm motility, spermatogenesis, and steroid hormone synthesis^3–5^. Spermatogenesis is one of the fundamental processes of sexual reproduction, during which diploid spermatogonial stem cells differentiate into haploid spermatids through meiosis and mature into functional spermatozoa. During this process, germ cells undergo drastic structural and functional changes that require constant adaptation to energetic needs, which is reflected by changes in mitochondrial morphology, in the stage-specific expression of mitochondrial proteins and a metabolic shift from glycolysis to oxidative phosphorylation (OXPHOS) during meiosis^6,7^. In addition to their role in energy supply, mitochondria play an important role during steroid hormone synthesis^8^. Regulated by luteinizing hormone (LH), a gonadotropin released by the pituitary gland, Leydig cells in the testes produce testosterone from cholesterol. Testosterone synthesis starts at the mitochondria where the first catalytic step takes place, during which cholesterol is synthesized to pregnenolone, which is then transported to the smooth endoplasmic reticulum (sER), where the testosterone synthesis is finalized. While other pathways exist, the canonical testosterone synthesis pathway is a multistep process, wherein the final step androstenedione is catalyzed to testosterone by HSD17B3. During steroid hormone synthesis mitochondria and sER work together in close concert, both organelles being connected via mitochondrial-associated membranes (MAM) in the ER. These MAMs possess a unique protein composition and are tethered to mitochondria via MFN2 that builds homo- and heterotypic complexes with MFN2 and MFN1 in the mitochondrial membrane^9^. The importance of mitochondria during steroid hormone synthesis is reflected through the huge number of mitochondria in Leydig cells and the influence of mitochondrial morphology on stereogenic function. Mitochondria are dynamic organelles that frequently undergo fusion and fission, which influences stereogenic capacity^10^. Testosterone is essential for the regulation of spermatogenesis, and dysfunctions in testosterone synthesis are a common cause of male infertility^5^. The demanding processes of spermatogenesis and steroid hormone synthesis leave germ cells and Leydig cells especially vulnerable to mitochondrial defects, but the underlying mechanisms leading to spermatogenic arrest caused by mitochondrial dysfunction are not yet fully understood.

Through its diverse range of substrates, PARL, a mitochondrial protease located in the inner mitochondrial membrane (IMM), plays an important, yet controversially discussed, role in the maintenance of several mitochondrial functions, such as the regulation of apoptosis, mitophagy, mitochondrial morphology, and respiratory chain function, and is associated with human diseases such as type 2 diabetes and Parkinson’s disease^11–18^. PARL deficiency in mice leads to Leigh syndrome-like neuronal degeneration accompanied by progressive multiorgan atrophy starting at the age of 6 weeks and leading to death after 8 to 10 weeks^13,17^. A very similar phenotype is found in mice with a conditional knockout of PARL in the nervous system. Besides a delay in lethality of 4 weeks, mice with only a neuronal deficiency of PARL show the same multiorgan atrophy as full knockouts do, suggesting a neuronal origin of the phenotype. While for a long time the involvement of PARL in apoptosis was suggested as the major cause of the severe phenotype of PARL-deficient mice, more recent studies have found a deficit in respiratory chain function to be responsible for the neuronal degeneration. Through its substrate TTC19, PARL is involved in the maintenance of respiratory chain complex III and is also involved in the coenzyme Q synthesis, which is important for respiratory chain function^13^. In addition to the neuronal phenotype, PARL-deficient mice are infertile, with females showing atrophy of the uteri and males showing a decrease in testis size^17^. Interestingly, the gonadal phenotype is the only one that does not occur in mice with a conditional knockout of PARL in the nervous system^13^, raising the question how PARL is connected to male infertility.

Here, we show that PARL deficiency leads to the complete arrest of spermatogenesis at the stage of primary spermatocytes that is likely to be caused by severe additional defects in respiratory chain complex IV, leading to an ATP deficit. In addition, PARL-deficient mice show a drastic decrease in the expression of HSD17B3, a protein of the testosterone synthesis pathway that is located at the sER. While testosterone synthesis can be recovered by a bypass synthesis pathway, the absence of the canonical synthesis leads to dysregulation of LH production in the pituitary gland and, consequently, of downstream responses. Leydig cell mitochondria show severe morphological changes and a significant decrease in MFN1 expression, which might lead to a reduction of mitochondrial fusion and might link the mitochondrial effects caused by PARL deficiency to the sER.

## Results

### PARL deficiency leads to histological abnormalities in the seminiferous tubules and arrest of spermatogenesis during meiosis I

**Fig. 1:**
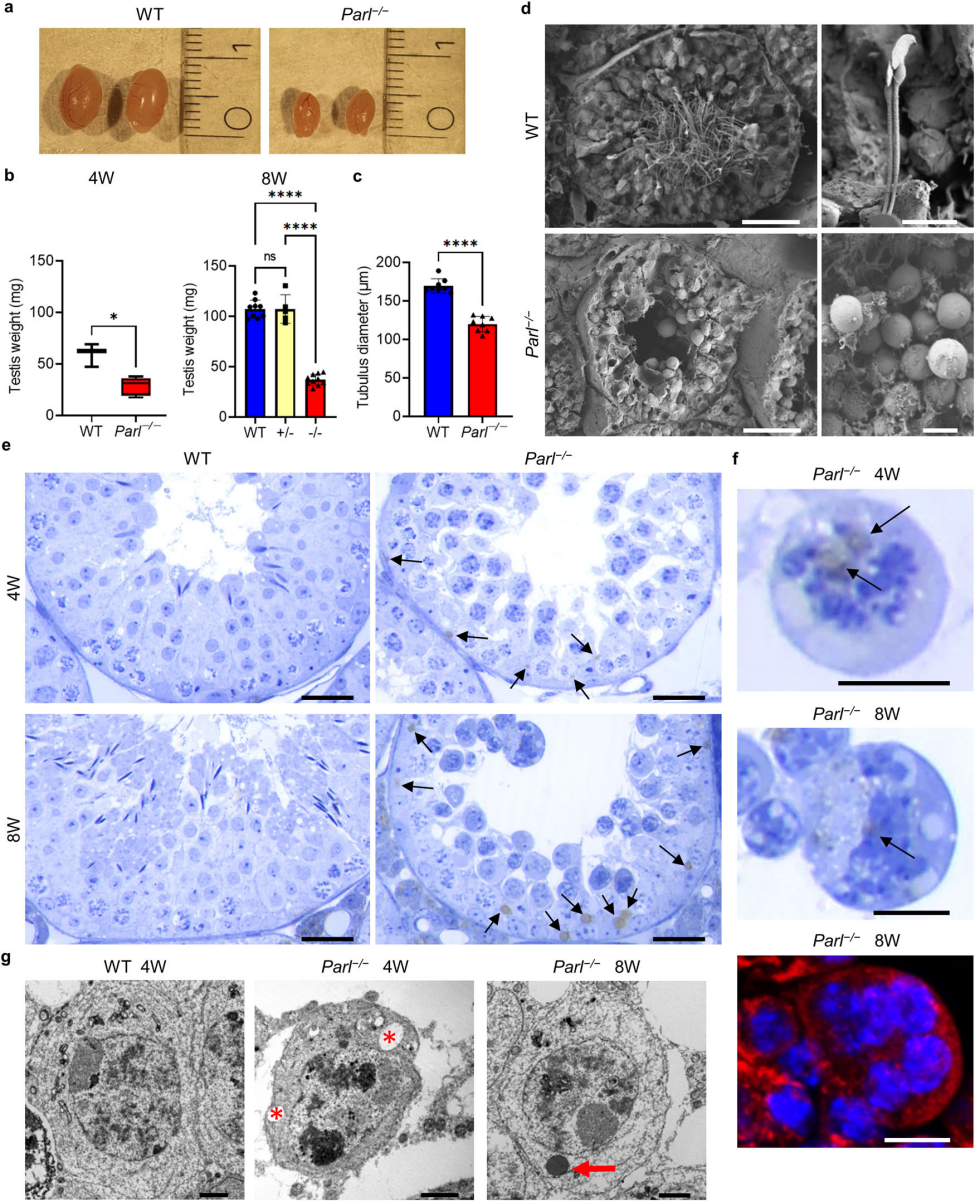
PARL deficiency leads to a reduction in testis weight and morphological degradation. **a** Testes of 8-week-old wild-type (WT) and PARL-deficient (*Parl*^−/−^) mice. **b** Average weight of both testes of 4-week- (4 W) and 8-week- (8 W) old mice with the indicated genotype. (4 W: WT: *n* = 3, *Parl*^−/−^: *n* = 5; 8 W: WT: *n* = 9, +/−: *n* = 5, −/−: *n* = 10; Error bars indicate standard deviation. Statistics: 4 W: Mann–Whitney *U* test: *p* = 0.0357, 8 W: Kolmogorov–Smirnov test *p* > 0.1000 for all groups, one-way ANOVA: *p* < 0.0001). **c** Average diameter of the seminiferous tubules of 8-week-old *Parl*^−/−^ mice in comparison to their WT litter mates. The average diameter of 10 tubules was taken for 8 animals (*n* = 8). (Error bars indicate standard deviation. Statistics: Kolmogorov–Smirnov test: WT: *p* = 0.0893, *Parl*^−/−^: *p* > 0.1000, unpaired two-tailed *t* test: *p* < 0.0001). **d** Scanning electron microscopy (SEM) pictures of seminiferous tubules of 8-week-old WT and *Parl*^−/−^ mice (scale bars; left: 50 µm, right: 10 µm). **e** Semi-thin sections of WT and *Parl*^−/−^ testes stained with toluidine blue at the age of 4 weeks and 8 weeks (scale bar: 25 µm). **f** Higher magnification of multinucleated giant cells in semi-thin sections and in immunohistological fluorescence staining (SDHA, nuclei were counterstained with bisbenzimide (blue)) of *Parl*^−/−^ mice at 4 and 8 weeks (scale bars: 10 µm). Arrows in (**e**) and (**f**) indicate lipid droplets within the seminiferous tubule and within the giant cells. **g** Transmission electron microscopy (TEM) pictures of spermatocytes of 4-week- and 8-week-old WT and *Parl*^−/−^ mice. Asterisks indicate vacuoles, and the arrow indicates a lipid droplet (scale bar: 2 µm).

**Fig. 2:**
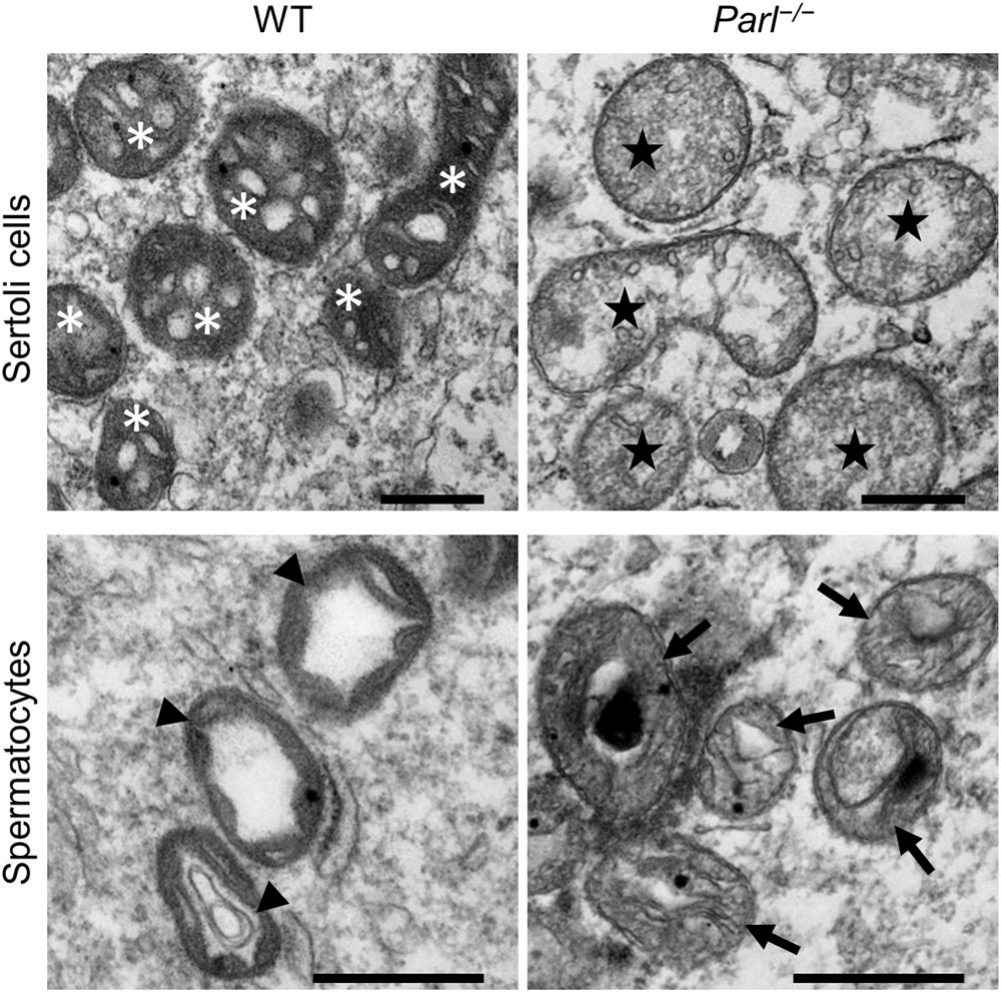
PARL deficiency leads to morphological defects in mitochondria. TEM pictures of mitochondria in Sertoli cells and spermatocytes of 8-week-old WT and *Parl*^−/−^ mice (scale bars: 500 nm). WT testes show physiological mitochondria with a clear intact cristae structure in Sertoli cells (asterisks) and the typical condensed type of mitochondria in spermatocytes (arrow heads). Mitochondria of *Parl*^−/−^ Sertoli cells (stars) and spermatocytes (arrows) show corruption of cristae morphology and are less electron dense. Furthermore, the mitochondria of spermatocytes display a reduction of the condensed type that is physiologically found in spermatocytes.

Testes of postpubertal *Parl*^−/−^ mice showed a macroscopically visual reduction in size (Fig. 1a) and a significant reduction in weight compared to their wild-type (WT) and heterozygote (*Parl*^*+/−*^) litter mates, which was already present at the age of 4 weeks, with an increased effect at an age of 8 weeks (Fig. 1b). While there is also a slight reduction in body weight in *Parl*^−/−^ mice at the age of 8 weeks, the reduction in testis size and weight can be best explained by a significant decrease in the diameter of the seminiferous tubules (Fig. 1c). The scanning electron microscopy (SEM) analyses, the microscopy of semi-thin sections and PAS-stained sections showed that there were neither spermatozoa nor round or elongated spermatids present in the tubules of *Parl*^−/−^ mice at any time point of investigation (P10, P20, 4 W and 8 W) (Fig. 1d, e, Fig. S1), suggesting a disruption of spermatogenesis during meiosis. Furthermore, semi-thin sections of the *Parl*^−/−^ testes showed increasing signs of degradation in luminal germ cells with vacuolization (asterisk Fig. 1g) and further histological abnormalities in the form of a high number of lipid droplets within the seminiferous tubules (arrows Fig. 1e–g) and multinucleated giant cells (Fig. 1f), already being present in 4-week-old *Parl*^−/−^ mice. Higher magnification as well as transmission electron microscopy (TEM) analysis showed that lipid droplets occurred within the Sertoli cells (Fig. S2) and even in the giant cells and normal spermatocytes (Fig. 1f, g). Similar multinucleated giant cells have been described in other studies showing spermatogenic arrest^19,20^ and might be a result of incorrect cell division but are more likely caused by cell fusion of degrading spermatocytes. Furthermore, multinucleated giant cells and lipid droplets have been described in aging mice^21^ and might be a sign of premature aging in *Parl*^−/−^ testes. The TEM images also revealed morphological abnormalities of the mitochondria in spermatocytes and in Sertoli cells with compromised cristae structure and reduction of the condensed-type mitochondria typically found in spermatocytes (Fig. 2). To determine whether cells in *Parl*^−/−^ testes undergo apoptosis, a TUNEL assay was performed, and immunohistological fluorescence staining as well as Western blots for caspase 3 were analyzed (Fig. S3). Both the TUNEL assay and the caspase 3 staining showed an increased number of apoptotic spermatocytes, and the Western blot confirmed a higher expression of caspase 3 from 4 weeks onward in *Parl*^−/−^ mice. However, not all spermatocytes underwent apoptosis within the seminiferous tubules, as demonstrated by a number of cells that entered and progressed through the epididymis (Fig. S4).

**Fig. 3:**
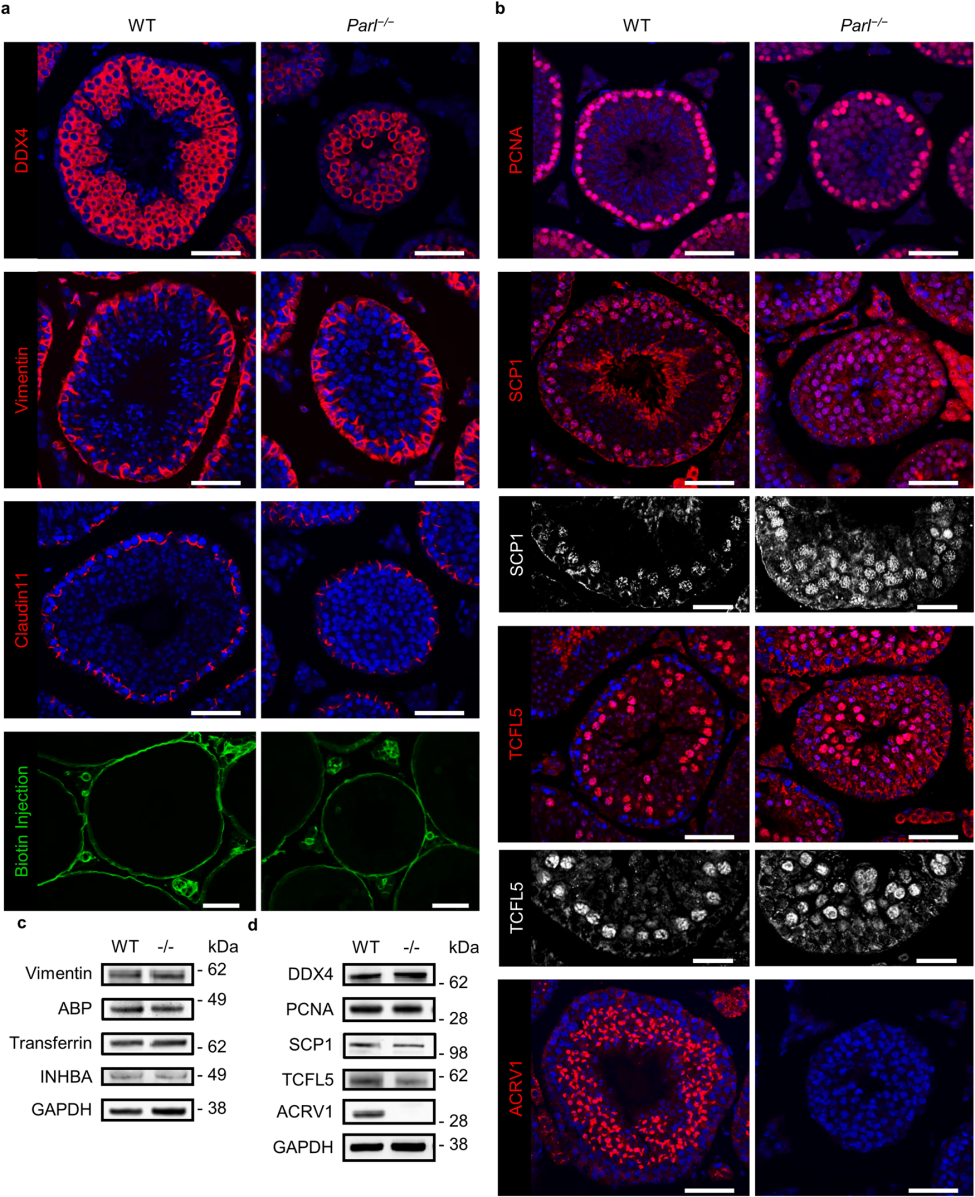
PARL deficiency leads to an arrest of spermatogenesis during meiosis I. **a** Immunohistological fluorescence staining against certain cell- and blood-testis-barrier marker proteins in testes of 8-week-old WT and *Parl*^−/−^ mice. DDX4: germ cells, vimentin: Sertoli cells, claudin 11 and biotin injection (a biotin tracer was injected into the interstitial space): blood-testis barrier (scale bar: 50 µm). **b** Immunohistological fluorescence staining in WT and *Parl*^−/−^ testes at the age of 8 weeks against germ cell stage marker proteins. PCNA: spermatogonial stem cells, SCP1: zygotene–early diplotene) spermatocytes, TCFL5: diplotene spermatocytes, ACRV1: spermatids (scale bar: 50 µm). For SCP1 and TCFL5 gray scale of the red channel is shown in higher magnification (scale bar: 25 µm). Nuclei were counterstained with bisbenzimide (blue). **c** Western blots of Sertoli cell proteins in testes of 8-week-old *Parl*^−/−^ mice in comparison to WT litter mates. GAPDH was used as an internal loading control. **d** Western blot of germ cell stage markers in WT and *Parl*^−/−^ testes at the age of 8 weeks. GAPDH was used as an internal loading control.

To evaluate the composition of the seminiferous tubules, further immunohistological fluorescence staining and Western blots for specific cell type- and stage marker proteins were analyzed (Fig. 3). The results suggested that there were no alterations in the number of Sertoli cells (Fig. 3a, vimentin staining), that the tight junctions of the blood-testis barrier (BTB) were intact (Fig. 3a, claudin 11), and confirmed that, generally, there were germ cells (Fig. 3a, DDX4) in the testes of *Parl*^−/−^ mice. An injection of a biotin tracer into the interstitial space further revealed the functional integrity of the BTB, showing that biotin did not enter into the tubular lumina in either WT or *Parl*^−/−^ animals (Fig. 3a). Further analyses of specific spermatogenic stage markers showed that the number of spermatogonial stem cells (PCNA staining) was unaltered in *Parl*^−/−^ mice (Fig. 3b). Positive staining with SCP1, a protein of the synaptonemal complex, showed that spermatocytes at least entered the zygotene stage of prophase I, where the transverse filaments of the synaptonemal complex first appear^22^. The presence of luminal cells that did not show the typical pachytene SCP1 staining (Fig. 3b) suggested a further progression into diplotene prophase. This was confirmed by the staining with TCFL5, a transcription factor that is specifically expressed in diplotene spermatocytes^20,23^, which showed that most luminal spermatocytes within the *Parl*^−/−^ tubules were TCFL5-positive. The disruption of spermatogenesis was then confirmed by the absence of staining with ACRV1, an acrosomal protein, which revealed the absence of spermatids in the tubules of *Parl*^−/−^ mice. Spermatocytes found in the epididymis were also stained with TCFL5 (Fig. S4), but no cells stained with SCP1 or ACRV1 could be found in the epididymis, supporting the assumption that spermatogenesis was arrested at the stage of diplotene prophase I and that spermatocytes were released from the tubules at this stage. The results of the immunohistological staining were confirmed by Western blots, which showed no changes in the expression of vimentin as well as ABP, transferrin and INHBA, proteins secreted by Sertoli cells, suggesting that, in addition to the intact BTB, general secretion function of Sertoli cells was intact. Furthermore, DDX4, PCNA, and SCP1 were expressed on similar levels in WT and *Parl*^−/−^ mice, while Western blots showed a slight decreased expression of TCFL5 and a complete absence of ACRV1 (Fig. 3c). Together, these results show severe anatomical abnormalities as early as in 4 week-old *Parl*^−/−^ mice and show a complete spermatogenic arrest within meiosis I, probably at the stage of diplotene spermatocytes, while the BTB and secretion function of Sertoli cells seemed to be intact.

### PARL deficiency causes an additional defect in respiratory chain complex IV found only in testes, leading to a decrease in complex IV activity and an ATP deficit

**Fig. 4:**
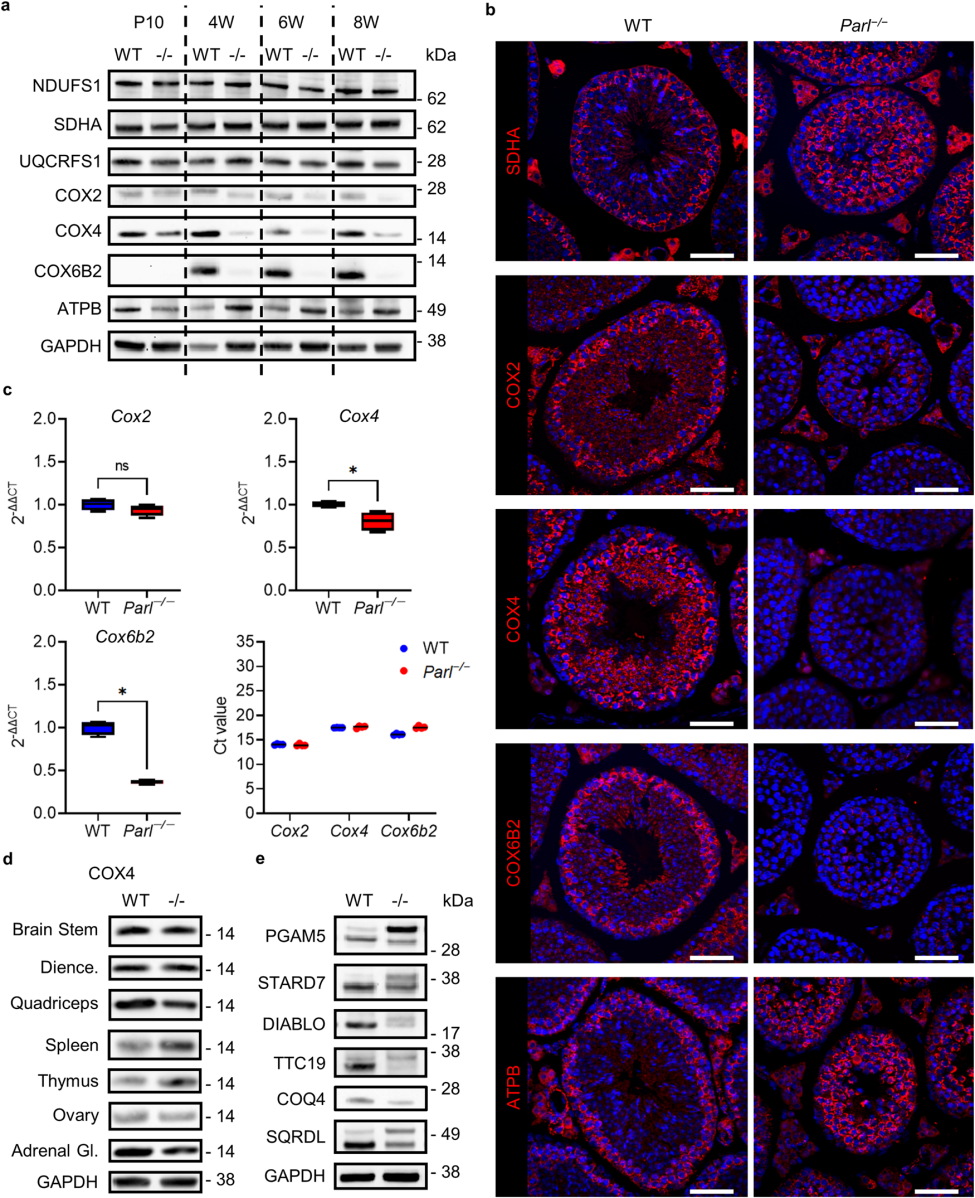
PARL deficiency causes testis-specific downregulation of respiratory chain complex IV proteins. **a** Western blots of respiratory chain complex I-V proteins in WT and *Parl*^−/−^ testes at the age of 10 days (P10), 4 weeks (4W), 6 weeks (6W), and 8 weeks (8W). NDUFS1: complex I, SDHA: complex II, UQCRFS1: complex III, COX2, COX4, and COX6B2: complex IV, ATPB: complex V. GAPDH was used as a loading control. **b** Immunohistological fluorescence staining against proteins of respiratory chain complex II, IV, and V of 8-week-old WT and *Parl*^−/−^ testes. Nuclei were counterstained with bisbenzimide (blue) (scale bar: 50 µm). **c** RT-qPCR 2^-ΔΔCT^ values and Ct values of 8-week-old *Parl*^−/−^ testes in comparison to WT for *Cox2*, *Cox4* and *Cox6b2* (*n* = 4). *Gapdh* was used as a reference gene and the average ΔCT value of the WT was used for calculating the ΔΔCT value. (Statistics: Mann–Whitney *U* test: *Cox2*: *p* = 0.3429, *Cox4*: *p* = 0.0286, *Cox6b2*: *p* = 0.0286). **d** Western blots of COX4 in 8-week-old WT and *Parl*^−/−^ mice in brain stem, diencephalon (Dience.), quadriceps, spleen, thymus, ovary, and adrenal gland (Adrenal Gl.). GAPDH was used as a loading control and is shown as an example of the diencephalon. **e** Western blots of a selection of PARL substrates and proteins affected by PARL deficiency in WT and *Parl*^−/−^ testes of 8-week-old mice.

**Fig. 5:**
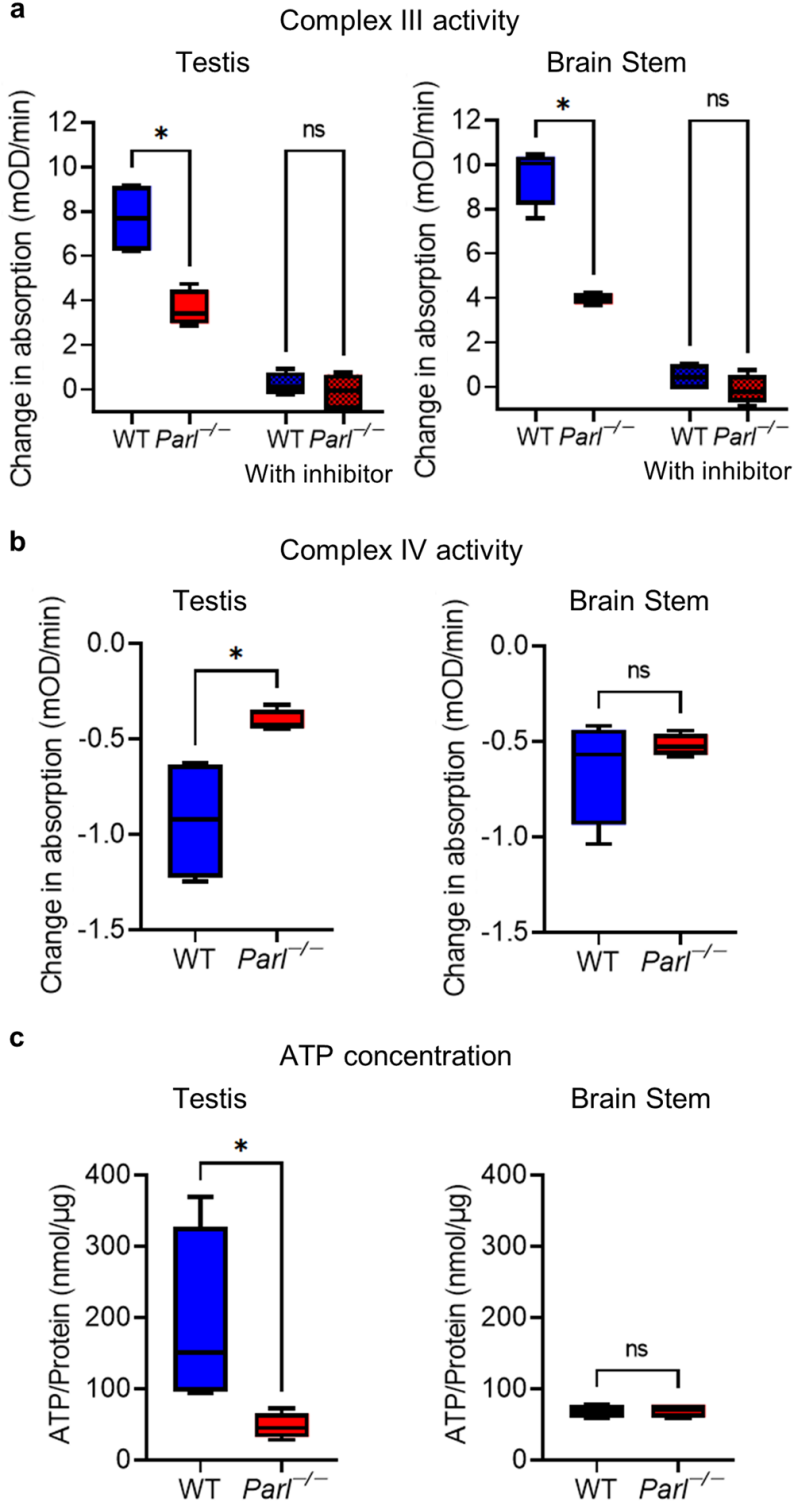
PARL deficiency leads to a testis-specific decrease in complex IV function and a deficit in ATP production. **a** Complex III activity of isolated mitochondria from the testis and brain stem of 8-week-old WT and *Parl*^−/−^ mice (*n* = 4) without and with complex III inhibitor antimycin a. Activity was measured as the change in absorption over time due to oxidation of cytochrome c. (Statistics: Mann–Whitney *U* test: testis: without inhibitor: *p* = 0.0286, with inhibitor: *p* = 0.4857, brain stem: without inhibitor: *p* = 0.0286, with inhibitor: *p* = 0.1143). **b** Complex IV activity in testis and brain stem of 8-week-old WT and *Parl*^−/−^ mice (*n* = 4), measured as the reduction in absorption due to the oxidation of reduced cytochrome c. (Statistics: Mann–Whitney *U* test: testis: *p* = 0.0286, brain stem: *p* = 0.8857). **c** ATP concentration in testis and brain stem of 8-week-old WT and *Parl*^−/−^ mice (*n* = 4). (Statistics: Mann–Whitney *U* test: testis: *p* = 0.0286, brain stem: *p* > 0.9999).

Respiratory chain function is crucial for the correct onset of spermatogenesis. During spermatogenesis, germ cells undergo a metabolic shift from glycolysis to OXPHOS, with spermatocytes showing the highest expression of respiratory chain proteins^6^. Because PARL is essential in the maintenance of correct respiratory function^13^, we set out to investigate the expression of certain subunit proteins of respiratory chain complexes I-V at different ages (Fig. 4a–c). While the proteins of complexes I-III and complex IV showed no obvious changes in expression in Western blots, all three tested proteins of complex IV (COX2, COX4, and COX6B2) were severely downregulated from 4 weeks onward in *Parl*^−/−^ testes (Fig. 4a, Fig. S5). The effect was most severe on COX6B2, a testis-specific protein isoform of complex IV, which was almost completely missing in *Parl*^−/−^ testes. Interestingly, no downregulation of COX4 was detectable in other tested organs, indicating that the defect of complex IV could be a testis-specific phenotype occurring only with onset of meiosis (Fig. 4d). Known PARL substrates and proteins affected by PARL deficiency also showed alterations in expression in *Parl*^−/−^ testes (Fig. 4e). Of special interest are TTC19, a protein that regulates complex III assembly, and COQ4, a protein involved in coenzyme Q synthesis that is important for respiratory chain function. Both were also downregulated in *Parl*^−/−^ testes, suggesting that the defect of complex III previously described in the brain also exists in the testis. Furthermore, the results of immunohistological fluorescence staining confirmed the highest expression of respiratory chain proteins in spermatocytes and the reduction of COX2, COX4, and COX6B2 in germ cells of *Parl*^−/−^ mice, with staining mostly restricted to the Leydig cells (Fig. 4b). With spermatocytes being highly dependent on OXPHOS as a source of ATP, this defect is a reasonable explanation for spermatogenic arrest in spermatocytes. Indeed, measurements of complex III and complex IV activity as well as ATP concentration in *Parl*^−/−^ testes compared to their WT litter mates confirmed a decrease in functionality of both complexes and a deficit in ATP production (Fig. 5). Interestingly, the measurements of complex III and IV activity and ATP concentration in the brain stem, a heavily affected part of the brain in *Parl*^−/−^ mice, showed only a decrease in complex III activity, while there was no significant decrease in function of complex IV or ATP concentration (Fig. 5), highlighting the influence of the additional defects in respiratory chain complex IV in the testes.

To obtain further insight into whether the loss of the proteins shown in Western blots was due to reduced transcription or whether the effect was translational/post-translational, we performed RT-qPCRs for *Cox2* (mitochondrial encoded), *Cox4* and *Cox6b2* (both nuclear encoded) (Fig. 4c). The results show no change in the transcription of *Cox2*, a small but significant decrease (~20%) in the transcription of *Cox4* (2^-ΔΔCT^ < 1), and a more substantial decrease in the transcription of *Cox6b2* with mRNA levels dropping to around 36.5% of that in WT littermates. This suggests that the loss of complex IV proteins cannot be accounted for solely by missing gene transcription and indicates an additional translational or post-translational effect leading to the loss of proteins. Together, these results show an additional downstream defect in respiratory chain complex IV caused by PARL deficiency that seems to be unique to the testis and is a likely explanation for the disruption of spermatogenesis at the stage of diplotene spermatocytes due to ATP insufficiency.

### PARL deficiency causes disruption of canonical testosterone synthesis, leading to a dysbalance in luteinizing hormone (LH) regulation

**Fig. 6:**
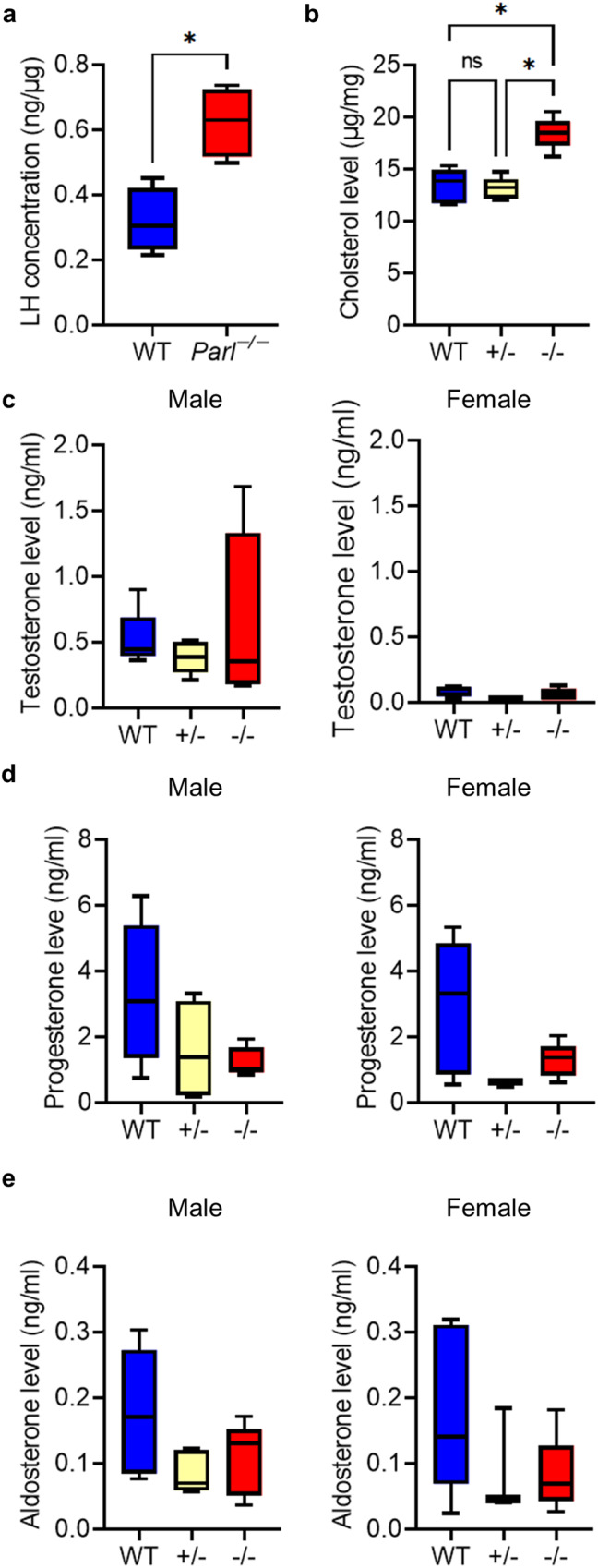
PARL deficiency leads to an increase of the LH and cholesterol level but steroid hormone levels remain unaltered. **a** Concentration of luteinizing hormone (LH) relative to the general protein concentration in the pituitary gland of WT and *Parl*^−/−^ mice (*n* = 4). (Statistics: Mann–Whitney *U* test: *p* = 0.0286). **b** Cholesterol level per mg testis of mice with the indicated genotype (*n* = 5). (Statistics: Kruskal–Wallis test: *p* = 0.0029). **c** Testosterone level in blood serum of male and female mice with the indicated genotype (*n* = 5). (Statistics: Kruskal–Wallis test: male: *p* = 0.7945, female: *p* = 0.157). **d** Progesterone level in blood serum of male and female mice with the indicated genotype (*n* = 5). (Statistics: Kruskal–Wallis test: male: *p* = 0.2391, female: *p* = 0.1389). **e** Aldosterone level in blood serum of male and female mice with the indicated genotype (*n* = 5). (Statistics: Kruskal–Wallis test: male: *p* = 0.1646, female: *p* = 0.5495).

**Fig. 7:**
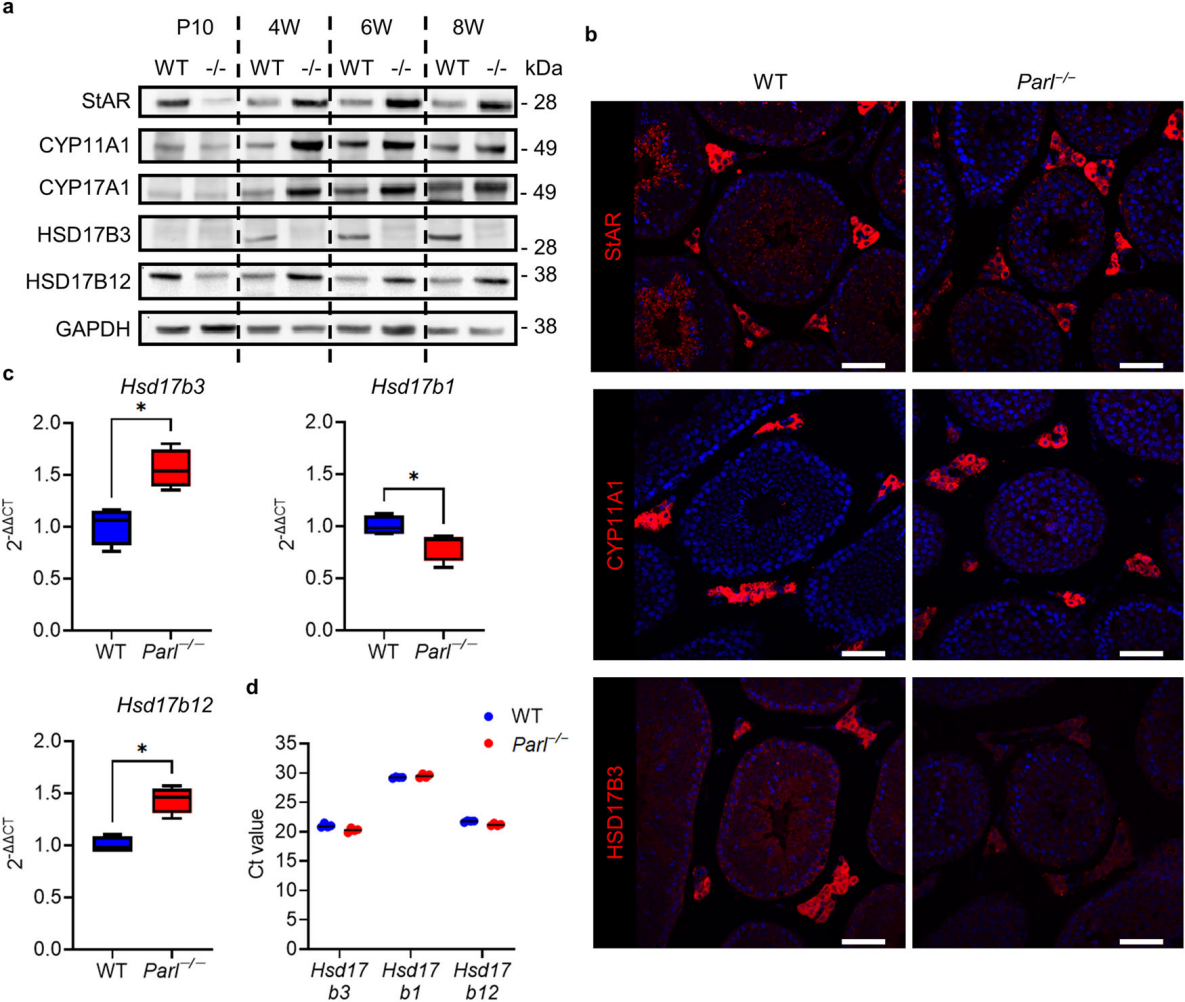
PARL deficiency leads to disruption in canonical testosterone synthesis by loss of HSD17B3 that is covered by the overexpression of HSD17B12 and an upregulation of LH regulated proteins. **a** Western blots of proteins of testosterone synthesis in the testes at 10 days, 4 weeks, 6 weeks, and 8 weeks in WT and *Parl*^−/−^ mice. GAPDH was used as a loading control. **b** Immunohistological fluorescence staining of selected proteins of the testosterone synthesis of WT and *Parl*^−/−^ testis at the age of 8 weeks. Nuclei were counterstained with bisbenzimide (blue) (scale bar: 50 µm). **c** RT-qPCR 2^-ΔΔCT^ values of *Hsd17b3*, *Hsd17b1* and *Hsd17b12* in testes of 8-week-old *Parl*^−/−^ mice in comparison to WT (*n* = 4). *Gapdh* was used as a reference gene. Statistics: *Hsd17b3*, *HSD17b1*, *Hsd17b12*: Mann–Whitney *U* test: *p* = 0.0286. **d** Mean Ct values of the triplicates for each sample of the qPCR for *Hsd17b3*, *HSD17b1*, *Hsd17b12*. The black line indicates the median of each group.

Testosterone is an important hormonal regulator of spermatogenesis, and deficits in testosterone lead to subfertility or complete infertility^5^. Testosterone becomes a regulatory factor after spermatocytes have crossed the BTB and is essential for initiating complete spermatogenesis during puberty and maintaining its continuance. Since *Parl*^−/−^ mice did not show signs of complete spermatogenesis at any age and because effects on the respiratory chain only occurred with the onset of puberty, we investigated whether there are distortions in hormonal regulation that might interfere with spermatogenesis or even puberty. To obtain an insight into the hormonal status of *Parl*^−/−^ mice, we measured the LH, cholesterol, and testosterone levels of 8-week-old male *Parl*^−/−^ mice in comparison to their WT and *Parl*^*+/−*^ litter mates. The results showed a significant increase in the LH level in the pituitary gland and in the cholesterol level in the testes of *Parl*^−/−^ mice (Fig. 6a, b). The testosterone level in the blood serum of *Parl*^−/−^ mice, however, was not significantly altered compared with WT mice (Fig. 6c). Additionally, neither male nor female *Parl*^−/−^ mice showed alterations in their serum levels of progesterone or aldosterone (Fig. 6d, e). Western blots of several proteins of the testosterone synthesis pathway allowed a closer look at the different steps of testosterone production (Fig. 7a). The mitochondrial proteins StAR (cholesterol transport to the inner mitochondrial membrane) and CYP11A1 as well as CYP17A1, located in the sER, were slightly upregulated in *Parl*^−/−^ mice from 4 weeks onward. The expression of these proteins is stimulated by LH, so the increase in expression could be explained by the elevated LH level. Surprisingly, HSD17B3, the protein that catalyzes the last synthesis step from androstenedione to testosterone in the canonical testosterone synthesis pathway, was almost absent in *Parl*^−/−^ mice at all tested ages. This was confirmed by the immunohistological data of 8-week-old mice, which showed almost no staining in *Parl*^−/−^ testes for HSD17B3 (Fig. 7b).

To elucidate further on the nature of the defect in HSD17B3 expression, we performed RT-qPCR of *Hsd17b3* (Fig. 7c). In contrast to the results on protein level, the RT-qPCR results showed a significant increase in *Hsd17b3* transcription in *Parl*^−/−^ testes (2^-ΔΔCT^ > 1). This result suggests that the loss of HSD17B3 was due to translational or post-translational defects rather than transcriptional defects. The increased transcription of *Hsd17b3* could be explained by its stimulation by LH and the elevated LH level. Since serum testosterone levels were normal, it is conceivable that an alternative pathway exists that leads to testosterone that is employed in *Parl*^−/−^ mice. On the one hand, HSD17B1 could be a possible candidate as it is involved in the production of testosterone in the Sertoli cells of prenatal mice and on the other hand HSD17B12 has been suggested as a possible alternative to perform the final step in testosterone synthesis in HSD17B3-deficient mice^24^. Our data show that HSD17B1 can be excluded as a possible bypass protein in *Parl*^−/−^ mice. The expression of *Hsd17b1* on the mRNA level was slightly but significantly decreased in *Parl*^−/−^ testes. CT-values between 29.05 and 29.86, furthermore, indicate that mRNA levels were generally very low in both genotypes (Fig. 7c, d). However, Western blot as well as qPCR data for *Hsd17b12* showed an increase in expression at both the mRNA and the protein level in *Parl*^−/−^ testes, making it a possible candidate to recover testosterone synthesis (Fig. 7a, c).

**Fig. 8:**
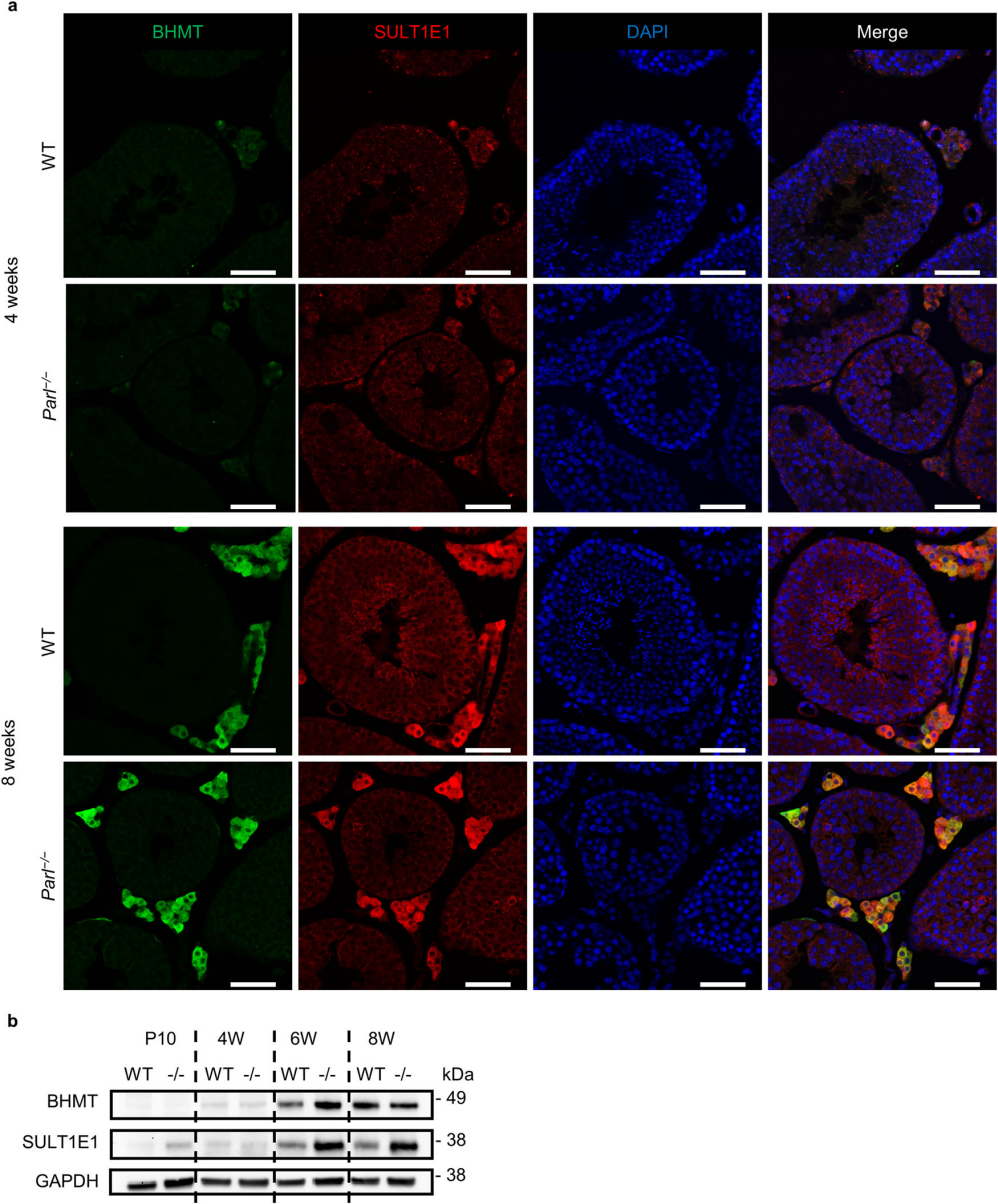
Postpubertal PARL deficient Leydig cells express ALC marker proteins. **a** Immunohistological fluorescence staining of two ALC marker proteins BHMT (green) and SULT1E1 (red) at the age of 4 and 8 weeks in WT and *Parl*^−/−^ testes. Nuclei were counterstained with bisbenzimide (blue) (scale bar: 50 µm). **b** Western blots of ALC marker proteins at the age of 10 days, 4 weeks, 6 weeks and 8 weeks in WT and *Parl*^−/−^ testes. GAPDH was used as a loading control.

Since HSD17B3 is only expressed in adult Leydig cells (ALCs), we wondered whether the severe reduction of HSD17B3 in *Parl*^−/−^ testes marks the absence of ALCs or whether ALCs exist but do not express HSD17B3. During the development of mammalian testes there are two distinct populations of Leydig cells that differ in morphology and function. Fetal Leydig cells (FLCs) exist during fetal and early postnatal live and are gradually replaced by ALCs during puberty. While FLCs produce androgens, they lack HSD17B3 and do not produce testosterone. Instead, testosterone production is covered by the Sertoli cells during the fetal period. To test whether *Parl*^−/−^ testes possess ALCs, we did fluorescence stainings and Western blots for two marker proteins (BHMT and SULT1E1) that, in the testis, are only expressed in ALCs^25^. The results of the fluorescence staining show that while in Leydig cells of 4-week-old mice there was only very slight staining for each of the markers in WT and *Parl*^−/−^ mice, Leydig cells of both genotypes showed a similar and clear staining for both proteins at the age of 8 weeks (Fig. 8a). These results were confirmed by the Western blots, showing in both WT and *Parl*^−/−^ testes that both markers were only slightly expressed up to 4 weeks and expression increased after puberty (6 W and 8 W). Expression in WT and *Parl*^−/−^ testes were on a similar level with even a slightly increased expression of SULT1E1 in *Parl*^−/−^ compared to WT mice (Fig. 8b). To further confirm that testosterone was produced by Leydig cells in *Parl*^−/−^ mice and not e.g. by the Sertoli cells as is the case in prenatal mice, we isolated Leydig cells of 8-week-old WT and *Parl*^−/−^ mice, cultured them for 48 h and stimulated them for 2 h with human chorionic gonadotropin (hCG). Our preliminary results show that both WT and *Parl*^−/−^ Leydig cells produced testosterone (WT: 6.55 ng/mL, *Parl*^−/−^: 6.06 ng/mL per 1.5 × 10^5^ cells) in isolation and reacted with an increase of testosterone production upon hCG stimulation (WT: 29.71 ng/mL, *Parl*^−/−^: 24.54 ng/mL per 1.5 × 10^5^ cells). Together the results suggest that ALCs exist in *Parl*^−/−^ testes and are capable of testosterone production besides missing HSD17B3.

**Fig. 9:**
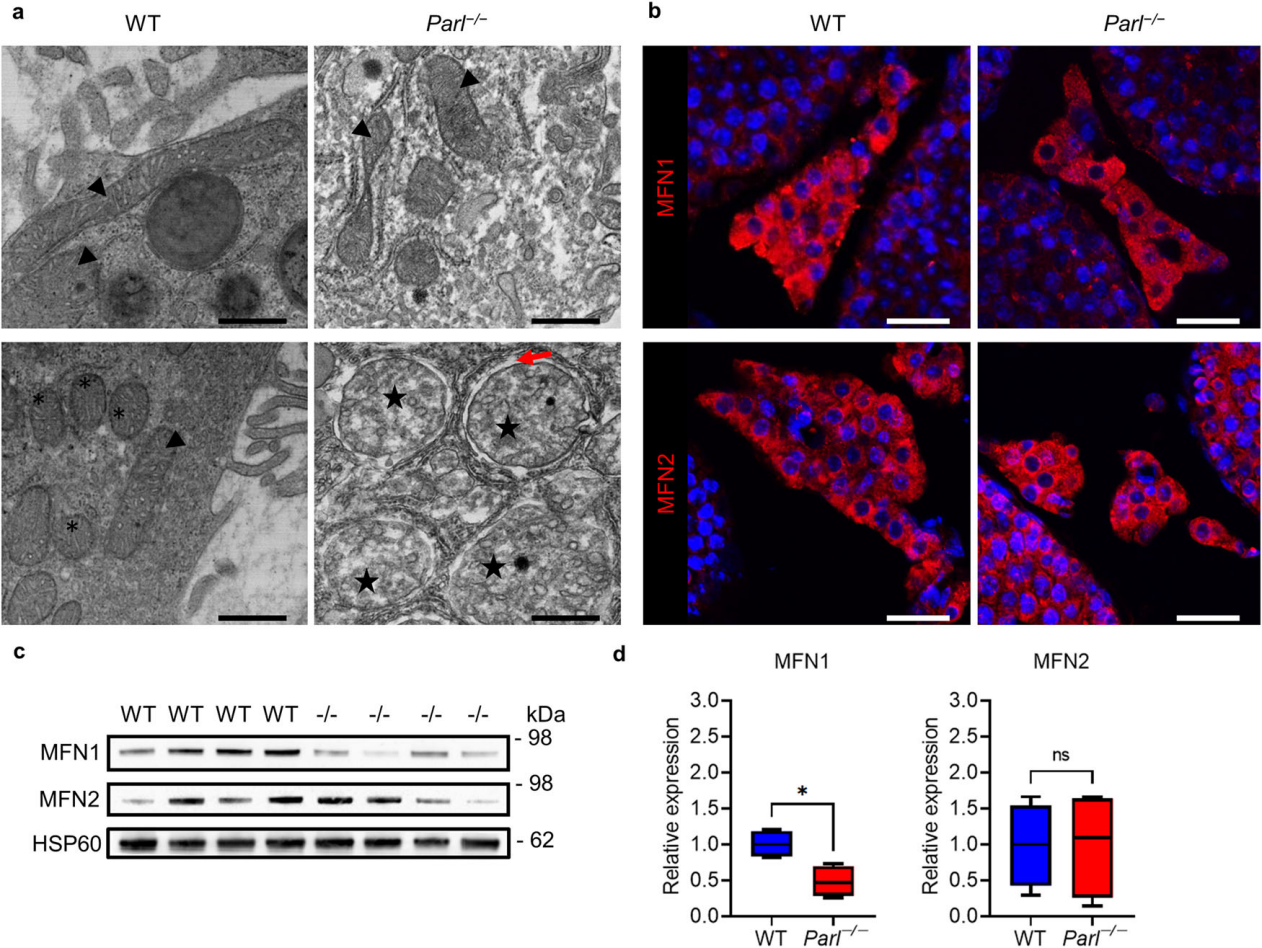
PARL deficiency leads to morphological changes in Leydig cell mitochondria and a downregulation of MFN1. **a** TEM pictures of mitochondria in Leydig cells of 8-week-old WT and *Parl*^−/−^ mice. For each genotype two examples are shown. Physiological elongated mitochondria are marked with arrow heads, physiological fragmented spherical mitochondria are marked with asterisks and degraded mitochondria with an increased diameter and compromised cristae structure are marked with stars. The red arrow marks the pronounced gap surrounding the degraded mitochondria (scale bars: 500 nm). **b** Immunohistological fluorescence staining of MFN1 and MFN2 in Leydig cells of 8-week-old WT and *Parl*^−/−^ mice (scale bar: 25 µm). **c** Western blots of MFN1 and MFN2 in isolated mitochondria of 8-week-old WT and *Parl*^−/−^ mice. HSP60 was used as a loading control. **d** Relative expression of MFN1 and MFN2 based on the Western blots. Graphical density values of the target protein were divided by the values of the loading control (HSP60) and then divided by the median value of the WT control group. Statistics: Mann–Whitney *U* test: MFN1: *p* = 0.0286, MFN2: *p* = 0.8857.

To get further insights into the effects of PARL deficiency on the ultrastructure of Leydig cells and specifically their mitochondria we used transmission electron microscopy (Fig. 9a, Fig. S2). In WT testes we observed the physiological typical characteristics of Leydig cells with a high number of elongated fused (Fig. 9a, arrow heads) and lower numbers of spherical fragmented (Fig. 9a, asterisks) mitochondria with an intact cristae structure. Elongated mitochondria were also found in *Parl*^−/−^ Leydig cells but less frequently. Instead, we observed high numbers of big inflated spherical mitochondria (Fig. 9a, stars) with compromised cristae structures that were less electron dense than the ones found in the WT cells. Furthermore, there was a noticeable gap around the mitochondria in the *Parl*^−/−^ cells clearly separating the mitochondria from the surrounding ER (Fig. 9a, red arrow). To find out whether the observed changes of mitochondrial morphology can be explained by a change in the expression of mitofusins (MFN1 and MFN2), we performed Western blot analysis as well as fluorescence staining (Fig. 9b, c). While neither Western blots nor the staining displayed changes in the expression of MFN2 in *Parl*^−/−^ testes, the expression of MFN1 was significantly reduced. Furthermore the staining showed that MFN1 and 2 were mostly expressed in Leydig cells with only little staining also found in spermatocytes. MFN1 and MFN2 promote fusion of mitochondria and are also involved in the maintenance of contact sides between mitochondria and the ER via MAMs^9,26^, thus the downregulation of MFN1 might explain the reduced number of elongated mitochondria and might influence the disassociation of mitochondria and ER.

**Fig. 10:**
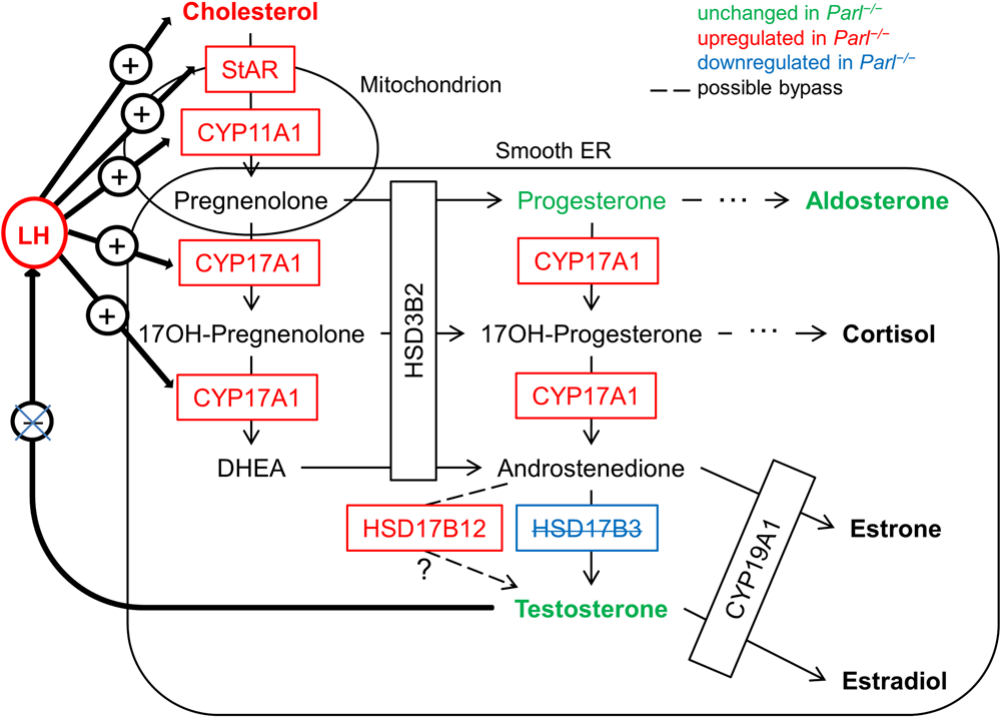
Graphical illustration of the effects of PARL deficiency on the testosterone synthesis pathway and LH regulation. Red indicates proteins and hormones that are upregulated in *Parl*^−/−^ mice, blue indicates downregulation in *Parl*^−/−^ mice, and green indicates measured hormones that show no significant difference between *Parl*^−/−^ and WT mice.

Taken together, these data (summarized in Fig. 10) show not only that deficiency of PARL, a mitochondrial protein, somehow leads to drastic downregulation of a protein critical for testosterone synthesis in the sER but also that there is an effective bypass pathway within the Leydig cells that leads to testosterone in the absence of the canonical pathway. However, the distortions in testosterone synthesis lead to dysregulation of the LH level and, consequently, all downstream responses. Downregulation of MFN1 might be a possible link between mitochondrial defects caused py PARL deficiency and the sER by compromising tethering of mitochondria and MAMs.

## Discussion

In this study, we show that the mitochondrial protease PARL plays an important role in the maintenance of spermatogenesis and the regulation of testosterone synthesis. PARL deficiency leads to a severe testicular phenotype with complete arrest of spermatogenesis at the stage of primary spermatocytes, probably in the diplotene prophase I, accompanied by cellular degradation of arrested cells (Figs. 1 and 3). This phenotype is probably a result of defects in respiratory chain function due to PARL deficiency. PARL is described to affect the assembly of complex III through its substrate TTC19 and to regulate coenzyme Q synthesis through COQ4, causing respiratory chain deficits leading to Leigh syndrome-like neuronal degeneration^13^. Comparable to the published data on the brain, TTC19 as well as COQ4 were downregulated leading to a significant decrease in complex III function in the testes of *Parl*^−/−^ mice (Figs. 4e, 5a). Importantly, our results indicate an additional deficiency of several proteins of complex IV that might be unique to the testis and was not found in other tested organs (Fig. 4). Measurements of complex IV activity and ATP concentration confirmed a decrease in complex IV function and a deficit in ATP production that was specific to *Parl*^−/−^ testes and did not occur in the brain stem, an organ heavily affected by PARL deficiency (Fig. 5b, c). Spermatogenesis and, specifically, meiosis are processes with high energetic costs that leave germ cells especially vulnerable to mitochondrial diseases that affect respiratory chain function. During spermatogenesis, germ cells undergo a metabolic shift from glycolysis toward OXPHOS as a method of ATP production^6^. Due to the drastic defects in respiratory chain function in *Parl*^−/−^ testes, the shift toward OXPHOS might lead to insufficient ATP production to cover the increasing energetic costs during meiosis that finally leads to an arrest of spermatogenesis in diplotene spermatocytes. However, to assess respiratory chain function in greater detail, further research using methods such as oxygraph or seahorse assays in isolated spermatocytes are needed.

Our results align with other studies that have reported spermatogenic arrest due to defects in mitochondria and their functions, such as ATP transport^27,28^, mitochondrial DNA^29^, or mitochondrial dynamics^30^. Of the tested organs the defect in respiratory chain complex IV was specific to the testes of *Parl*^−/−^ mice. Other organs that are heavily affected by the deficiency in PARL, such as the brain, muscles, spleen, and thymus, as well as other steroidogenic organs, such as the adrenal glands and ovaries (which is also an organ of germ cell maturation), did not show similar effects on complex IV protein expression due to PARL deficiency (Fig. 4d). Even within the testis, the defect seemed to be restricted to germ cells, with Leydig cells showing slight immunostaining against COX2 and COX4 (Fig. 4b). This highlights the importance of cell-specific downstream responses on mitochondrial deficiencies leading to specific phenotypes in different cell types. Germ cells are inherently different from other cells as they are the only cells to undergo meiosis, leading to very specific demands on general cell functions. The unique requirements of spermatogenesis are reflected through the existence of testis-specific isoforms of several proteins related to energy metabolism, such as proteins of glycolysis (e.g., PGK2 and LDHC)^31,32^, respiratory chain (Cyt c_t_, COX6B2)^33,34^, and ATP transport (ANT4)^27^, which are specifically expressed in spermatocytes entering meiosis as well as through the existence of a germ-cell-specific ribosome^35^.

So far little is known about how the shift from glycolysis towards OXPHOS is regulated during spermatogenesis and what triggers the expression of testis-specific proteins. Interestingly testis-specific proteins are often also expressed by cancer cells and are therefore also called cancer/testis antigens (CT antigens)^36^. The testis-specific protein of respiratory chain complex IV, COX6B2, is such a CT antigen and could be a possible factor linking the increase of OXPHOS during meiosis to the expression of testis-specific protein isoforms. So far the function of COX6B2 during spermatogenesis is poorly understood and studies in *Cox6b2* KO mice have mainly focused on the effect on sperm motility^37^. However, studies in pancreatic ductal cancer cells and lung adenocarcinoma cells have shown that the expression of COX6B2 in those cancer cells drives a very similar shift towards OXPHOS, increasing oxidative phosphorylation, leading to increased cell proliferation, promotion of metastasis and is associated with poor survival^38,39^. Knock down of COX6B2 in those cells led to a decrease of monomer complex IV, impaired complex IV assembly and a reduction of complex IV activity. Considering these results, COX6B2 might have a similar function in spermatocytes, promoting the shift towards OXPHOS. Our results show that COX6B2 was not expressed in 10-day-old WT mice, an age before germ cells start undergoing meiosis, and that expression was highest in spermatocytes (Fig. 4a, b). Interestingly, of the tested proteins, the effect of PARL deficiency was most severe on COX6B2, leading to an almost complete loss on the protein level. Furthermore, the decrease of COX2 and COX4 in *Parl*^−/−^ testes might be linked to the loss of COX6B2 as both proteins seem to be expressed normally in 10-day-old *Parl*^−/−^ testes and the decrease only occurs when COX6B2 is expressed in the WT but not in *Parl*^−/−^ animals. Together it is conceivable that the loss of COX6B2 in PARL-deficient mice might be the cause of the decrease in complex IV proteins and complex IV activity, similar to what was described before in cancer cells^38,39^. Additionally, the loss of testis-specific COX6B2 could offer an explanation as to why the defect of complex IV caused by PARL deficiency seems to be specific to the testis. Further studies, specifically in isolated spermatocytes, will have to be performed to obtain more insights into the processes underlying the defect of complex IV caused by PARL deficiency and the possible involvement of COX6B2. However, our results on the loss of COX6B2 in PARL deficient testes, a known CT antigen, show that understanding the mechanisms underlying cell-type-specific signaling of PARL might not only be essential for the treatment of infertility but might also have important implications beyond germ cells and fertility, opening new potential in cancer research.

Analyses of the transcription of *Cox2*,*Cox4* and *Cox6b2* showed that there was a significant decrease in the transcription of *Cox4* and *Cox6b2* in *Parl*^−/−^ testes, while *Cox2* transcription was unaltered (Fig. 4c). However, the reduction in transcription does not fully reflect the effect on the protein level, while *Cox6b2* mRNA levels in *Parl*^−/−^ testes were reduced to about 36.5% of that in WT testes, decrease on the protein level was much more severe with a loss of more than 95% of COX6B2 protein. Thus a pure transcriptional defect is unlikely. Further studies on the transcriptional profile of isolated spermatocytes at different ages will provide more information on the transcriptional effects of PARL deficiency.

Furthermore, our results showed that PARL plays a role in the regulation of testosterone synthesis (Figs. 6–10). PARL deficiency led to loss of the canonical testosterone synthesis pathway due to downregulation of HSD17B3 (Fig. 7). In the canonical testosterone synthesis pathway, HSD17B3 catalyzes the last step of testosterone synthesis, the conversion of androstenedione to testosterone. However, our results, along with the data from *Hsd17b3*^−/−^ mice^24^, showed that there is an effective alternative pathway in mice, leading to sufficient testosterone production. Other members of the HSD17B protein family, such as HSD17B1 and HSD17B12, are also able to catalyze androstenedione to testosterone, although with less efficiency^40–42^. HSD17B12 was suggested as the most promising candidate to take over testosterone synthesis as it is expressed in adult Leydig cells^24^. Our data (Fig. 7a, c) align with those from HSD17B3-deficient mice^24^ by showing an upregulation of HSD17B12 on the mRNA and protein level in the absence of HSD17B3, underlining its status as a potential bypass protein. HSD17B1 is another potential bypass protein, as it is responsible for testosterone production in the Sertoli cells of prenatal mice^43^. However, our data show that HSD17B1 is very unlikely to play a role in testosterone production in adult *Parl*^−/−^ testes. Similar to what is stated in the literature our results show that *Hsd17b1* is transcribed only on a very low level in adult mice and is even significantly reduced in PARL-deficient mice. Uncovering alternative testosterone synthesis pathways holds important potential for the treatment of human HSD17B3-deficient patients. In humans, testosterone production is not recovered in the absence of HSD17B3, which leads to more severe phenotypes, including pseudohermaphroditism and infertility^44^.

Testosterone plays an essential role in initiating spermatogenesis at the onset of puberty and maintaining its continuance from there on^45^. The production of testosterone is regulated in a complex positive and negative feedback loop through LH. Simply put, LH stimulates testosterone production, among other ways, through an increase in the expression of proteins involved in testosterone synthesis and an increased provision of cholesterol (through de novo synthesis and mainly through uptake of circulating LDL and HDL)^46^. Testosterone, in turn, inhibits LH production directly and indirectly through Kisspeptin (KiSS-1) and gonadotropin-releasing hormone (GnRH). Testosterone and estradiol (aromatized testosterone) inhibit the expression of Kisspeptin and its respective receptor (GPR54) in the hypothalamus. Kisspeptin usually induces the release of GnRH, which in turn stimulates LH production^47,48^. This hypothalamic-pituitary-gonadal (HPG) axis seems to be distorted somehow through the loss of normal testosterone synthesis, leading to an increased LH level, even though testosterone production is recovered through an alternative synthesis way (Figs. 7, 10). The upregulation of steroidogenic proteins as well as the increased cholesterol level in *Parl*^−/−^ testes are then probably the results of the elevated LH level (Fig. 6). A very similar effect was shown again in HSD17B3-deficient mice; *Hsd17b3*^−/−^ mice also showed an increase in LH, of proteins of testosterone synthesis, and additionally an increase in testosterone level^24,49^. Furthermore, mice with a hypomorphic allele of the androgen receptor gene (*Ar*), which leads to an AR deficiency with intact testosterone synthesis, also show a disruption of the HPG axis. These mice have significantly increased LH and testosterone levels, indicating the missing negative feedback of testosterone on LH, and show an increase in cholesterol concentration in Leydig cells and an upregulation of steroidogenic proteins^46^. However, the underlying mechanisms leading to the disruption of the HPG feedback system are not yet clear and require further investigation. Our data indicated that the deficiency of HSD17B3 cannot be explained by missing gene transcription, as the RT-qPCR results showed that there was a significant increase in transcription (Fig. 7c). Furthermore, the decrease of HSD17B3 was not due to missing adult Leydig cells. Our results show that PARL-deficient Leydig cells expressed ALC marker proteins (Fig. 8) and produced testosterone in isolated cell culture as indicated by our preliminary results, suggesting that ALCs exist and produce testosterone but do not express HSD17B3 on protein level. How the deficiency of PARL leads to a lack of HSD17B3 in the ER is still unknown and requires further investigation. So far, there are no known pathways linking PARL to the ER. Mitochondria and ER are in close physical and functional relation to each other and are connected through mitochondrial-associated membranes (MAM) within the ER. These MAMs possess a unique protein composition and are essential for cellular functions such as lipid transport/synthesis, Ca^2+^-signaling, mitochondrial dynamics and steroid hormone synthesis^50^. MFN1 and 2 are proteins of the outer mitochondrial membrane (OMM) that are essential for the regulation of mitochondrial fusion and are involved in the formation of contact sides between mitochondria and MAMs in the ER^9,26^. Our data on the morphology of PARL-deficient Leydig cells show degradation of numerous mitochondria in the form of compromised cristae morphology and a reduction of elongated/fused mitochondria suggesting a reduction of mitochondrial fusion (Fig. 9). Indeed our Western blot data demonstrated a significant reduction of MFN1 in *Parl*^−/−^ testis mitochondria (Fig. 9c), which might explain the reduced number of elongated mitochondria. Studies have shown that mitochondrial dynamics are directly linked to steroid hormone synthesis, showing that during testosterone production mitochondrial fusion is promoted and increased mitochondrial fission leads to a reduction in steroid hormone synthesis^10^. Furthermore, our TEM analysis might indicate a dissociation of mitochondria and ER (Fig. 9a, red arrow), which might also be influenced by the reduction of MFN1 and might link the mitochondrial defects caused by PARL deficiency to the sER and HSD17B3. Tethering between ER and mitochondria is achieved through coupling of MFN2 located in MAMs and MFN1 and 2 on the OMM^9^. While MFN2 is mainly responsible for juxtaposition of ER and mitochondria, the reduction of MFN1 might still influence tethering in some way. However, to get better insights into the interactions between PARL, MFN1 and HSD17B3 further studies in isolated Leydig cells are needed. Our results highlight the functional interactions between mitochondria and other organelles such as the ER and show that the consequences of mitochondrial defects can reach pathways outside the mitochondria. Insights into these functional interactions are crucial for our understanding of mitochondrial diseases and their systemic effects.

Another aspect that is worth further investigation is the functional link between Sertoli and germ cells as well as Sertoli and Leydig cells. Conditional knockout of AR in Sertoli cells leads to an arrest of spermatogenesis and a significant decrease in the testosterone level in adult mice, showing that Sertoli cell feedback is essential for both spermatogenesis and testosterone synthesis^51^. While mitochondria of PARL-deficient Sertoli cells also showed severe morphological degradation (Fig. 2), the BTB induced by Sertoli cells was intact and ABP, inhibin (INHBA) and transferrin were produced on a similar level as in WT mice (Fig. 4a, c). However, the influence of PARL deficiency on further aspects of Sertoli cell function, specifically on the provision of lactate for germ cells, needs to be investigated in more detail in future studies.

Crucially, our results demonstrate that PARL plays an important role in two essential aspects of spermatogenesis and male fertility by maintaining respiratory chain function and being involved in the regulation of testosterone synthesis. This study provides important insights into cell-type-specific responses to mitochondrial deficits and functional interactions between cell organelles that extend our understanding of male infertility and additionally hold exciting potential for research beyond fertility.

## Methods

### Mice

Full knockout mice with a germline deletion of *Parl* (*Parltm1.1Bdes* (*Parl*^−/−^) were provided by Prof. Bart De Strooper (KU Leuven) and generated as described by Cipolat et al.^17^. Mice were kept in small groups in a temperature- and humidity-controlled room with a 12-h light/dark cycle. Food and water were provided *ad libitum*. Breeding was performed using heterozygote (*Parl*^*+/−*^) animals. *Parl*^*+/+*^ animals were used as the wild-type (WT) control, and *Parl*^*+/−*^ animals were used in some experiments as an additional control. For tissues other than the testes, male and female animals were used. Animal handling was performed in strict accordance with Governmental Directive 2010/63/EU of the European Parliament and of the Council of September 22, 2010 further amended by regulation (EU) 2019/1010, institutional animal care regulations and ARRIVE guidelines.

### Western blot, RT-qPCR, ELISA, Complex III and IV activity and ATP detection

Animals at the age of 10 days (P10), 4 weeks (4 W), 6 weeks (6 W), and 8 weeks (8 W) were sacrificed using an overdose of carbon dioxide. Organs (testes/ovaries, brain [separated into brain stem, cerebellum, diencephalon, striatum, and cortex], pituitary gland, spleen, thymus, adrenal glands, and quadriceps) were dissected from the animals and snap-frozen in liquid nitrogen.

Western blotting tissues were homogenized in NP-40 buffer (50 mM Tris pH 7.4 (Merck), 150 mM NaCL, 1% Triton X-100 (Sigma Aldrich) and separated in Nupage 4%–12% Bis-Tris 10 well gels (Invitrogen, Thermo Fisher Scientific) at a protein concentration of 2 µg/µL. Proteins were transferred to Amersham Protran 0.45-µm nitrocellulose blotting membrane (Cytiva). Membranes were blocked for 1.5 h at room temperature (RT) with Roti-Block (Carl Roth) in PBS, incubated overnight at 4°C with the primary antibody in Roti-Block-PBS (for details see Table S1), washed with 0.05% Tween20 in PBS (TPBS), incubated for 1.5 h at RT with the secondary antibody (1:10,000 in Roti-Block-PBS with 0.5% milk, HRP goat anti-rabbit or mouse, Jackson ImmunoResearch), and washed with TPBS again. Amersham ECL Prime Western Blotting detection reagents (Cytiva) and a VersaDoc Imaging System MP 4000 (BioRad) were used to visualize protein bands.

For RNA isolation, a RNeasy Mini Kit (Qiagen) was used according to the instructions using 20 mg (±10%) of testis tissue from 8-week-old mice as the starting sample and including an on-column DNase step with a PureLink DNase Set (Thermo Fisher Scientific). Five hundred nanograms of RNA were reverse-transcribed to cDNA using a Maxima First Strand cDNA Synthesis Kit for RT-qPCR (Thermo Fisher Scientific), as described in the instructions, and included a reverse transcriptase minus control (RT-control). For the RT-qPCR, commercially available TaqMan Gene Expression Assays (Thermo Fisher Scientific) and TaqMan Fast Advanced Master Mix (Thermo Fisher Scientific) were used according to the manufacturer’s instructions with 1 µL of cDNA in 10 µL of reaction mix per reaction. The RT-qPCR was performed in triplicate for each sample in each gene using a LightCycler96 (Roche). As a negative control, cDNA was replaced by nuclease-free water for each gene.

The concentration of LH in the pituitary gland was measured using a Mouse LH Beta ELISA Kit (Abcam). A pituitary gland was disrupted and homogenized in 100 µL of the provided 1X Cell Extraction Buffer PTR, the protein concentration was measured using a Pierce BCA Protein Assay Kit (Thermo Fisher Scientific), and samples were used at a dilution of 1:400. Colorimetric results were measured using an Infinite M Plex (Tecan).

For the measurement of complex III and IV activity mitochondria were isolated from testis and brain stem using a mitochondria isolation kit for tissue (abcam). For WT a single testis (0.1 g ± 10%), for *Parl*^−/−^ both testes (0.07 g ± 10%) and 0.07 g ± 10% of brain stem of each animal were homogenized in 2 mL of the provided isolation buffer. The homogenate was first centrifuged at 4 °C with 1000 × *g* for 10 min and then the supernatant was centrifuged at 4 °C with 12,000 × *g* for 15 min. The resulting pellet was washed twice by resuspending in isolation buffer and centrifugation. For the measurement of complex III the pelleted mitochondria were resuspended in 35–70 µL isolation buffer and protein concentration was measured using a Pierce BCA Protein Assay (Thermo Fisher Scientific). The activity of respiratory chain complex III was measured using a Mitochondrial Complex III Activity Assay Kit (Sigma-Aldrich) according to the instructions by adding 5 and 10 µg of protein in 1–2 µL of mitochondrial sample to the reaction mix on the 96-well plate. Finally, 6 µL of cytochrome c was added to each well and the change in absorption was measured every 30 s for 10 min at wavelength of 550 nm. Samples were issued in duplicates for each concentration. For each sample, a control with complex III inhibitor was performed by adding 2 µL of antimycin a to the mix. Furthermore, a negative control was performed by adding 2 µL of water instead of a sample to the reaction mix. The change in absorption was calculated within the linear range, divided by the protein concentration and the average of both concentrations was calculated. For the measurement of complex IV activity we used a Complex IV Rodent Enzyme Activity Microplate Assay Kit (abcam) by resuspending the pelleted mitochondria in 35 µL (testis) or 75 µL (brain stem) of solution 1 provided in the kit, measuring the protein concentration using a Pierce BCA Protein Assay (Thermo Fisher Scientific), diluting the concentration to 5.5 µg/µL with solution 1 and adding 1:10 detergent (final concentration 5 µg/µL. After 30 min of incubation on ice, followed by centrifugation, 5 µL (25 µg protein) of the supernatant were loaded into the wells of the provided assay microplate with 195 µL of solution 1. Samples and null controls (only solution 1) were tested in triplicates. After 3 h of incubation at RT and washing with solution 1, 200 µL of Assay solution containing reduced cytochrome c were added to the wells and the decrease in absorbance at 550 nm wavelength was measured every 2 min for 2 hours at 30°C. The decrease in absorbance was calculated within the linear range of the measurement (between 10 and 44 min). Mitochondria that were isolated in excess were used for Western blotting in the same way as described above for tissue lysats.

The ATP concentration of the testis and brain stem were measured using a luminescence ATP detection assay kit (Cayman). Three hundred fifty milligrams of tissue sample was homogenized in 400 µL of the provided sample buffer and deproteinized by adding 200 µL of 1 M perchloric acid to a 200-µL aliquot of the sample, followed by neutralization with 1 M potassium hydroxide. Deproteinized samples were used undiluted and at a dilution of 1:5 following the kit instructions. Luminescence was measured using an Infinite M Plex (Tecan).

### Immunohistology and electron microscopy

Mice at the ages of 4 or 8 weeks were sacrificed by an overdose of carbon dioxide and post-mortem perfused through the left ventricle with phosphate-buffered saline (PBS), followed by perfusion with modified Bouin solution (4% paraformaldehyde [PFA], picric acid in PBS, pH 5, for immunohistology) or 6% glutaraldehyde in PBS (for electron microscopy). Testes were kept in the respective fixative overnight for post-fixation.

For immunohistological fluorescence staining, testes with epididymides were embedded in paraffin. Eight-micrometer-thick deparaffinized rehydrated sections were subjected to heat-mediated antigen retrieval with Tris-EDTA buffer (pH 9), blocked for 1.5 h at RT using 10% bovine serum albumin (BSA, Bio West), and incubated with the indicated primary antibody at 4 °C overnight (see Table S1 for details). After washing with PBS, slides were incubated for 1.5 h at RT with the secondary antibody (DyLight 549, Goat anti-Rabbit, Vector) and counterstained with bisbenzimide (H33258, Sigma). Fluorescence images were acquired using an Eclipse 90i microscope (Nikon) equipped with an Intensilight C-HGFIE fiber illuminator (Nikon) and a D-Eclipse C1 laser scanning confocal microscope system (Nikon). Pictures of WT and *Parl*^−/−^ were taken with the same parameters for each antibody.

To visualize apoptotic cells in paraffin-embedded testis sections, an ApopTag Peroxidase In Situ Apoptosis Detection Kit (Millipore), which applies the TUNEL method, was used according to the manufacturer’s instructions.

To test the functional integrity of the BTB, a biotin tracer (10 mg/mL EZ-Link Sulfo-NHS-LC-Biotin, Pierce) was injected into the interstitial space of the testes of freshly sacrificed mice, as described previously^52^. Injected testes were kept at RT for 30 min, and were then fixated in modified Bouin solution (pH 5) overnight and embedded in paraffin. To visualize the biotin tracer, 8-µm-thick deparaffinized rehydrated sections were blocked for 1 h with 5% BSA in PBS and incubated for 1 h at RT with DyLight 488 streptavidin (Vector).

For electron microscopy, testes were cut into slices measuring a few millimeters using a razor blade. The slices were washed in PBS, incubated for 1 h in 1% osmium tetraoxide (OsO_4_) in PBS, and dehydrated in an ethanol series. To prepare samples for scanning electron microscopy (SEM), slices were transferred to acetone using an increasing acetone series in ethanol, dried using a critical point dryer (CDP 030; Bal-Tec), fixed on a sample holder (sample holder for JEOL, Plano), and sputtered with platinum using a sputter-coater SCD 500 (Bal-Tec). Images were obtained using an SEM 7500 F (JEOL). For transmission electron microscopy (TEM), dehydrated samples were incubated in propylene oxide, followed by incubation and embedding in a SPURR medium. Semi-thin sections of 1-µm thickness were obtained and stained with toluidine blue. Ultra-thin sections were mounted on copper grids (200 G or 300 G mesh, Plano), contrasted, and analyzed using a JEM-1400 TEM (JEOL).

### Testis cholesterol and serum steroid hormone levels

The cholesterol levels of 10 mg of dried testis samples were measured by flame-ionization detection following gas chromatographic separation (GC-FID) on a DB-XLB 30 m × 0.25 mm i.d. × 0.25 µm film (J&W Scientific Alltech) in a Hewlett-Packard 6890 Series GC-system (Agilent Technologies) equipped with an FID at the Institute of Clinical Chemistry and Clinical Pharmacology of the University Hospital Bonn as described in a previous study^53^.

For the measurement of steroid hormones, blood samples of 8-week-old mice were obtained postmortem (mice sacrificed for organ harvesting) through intracardial extraction from the right ventricle. Steroid hormones were determined at the Institute of Laboratory Medicine, Clinical Chemistry and Molecular Diagnostics, University of Leipzig (Leipzig, Germany). 100 µL of serum was used for the simultaneous quantification of aldosterone, testosterone and progesterone using online solid phase extraction (SPE) LC-MS/MS as described in a previous study^54^. Lower limits of quantification ranged from 0.02 ng/mL (aldosterone), 0.03 ng/mL (testosterone) to 0.06 ng/mL (progesterone). General accuracy was 95–109% with between-run imprecision of ≤10%.

### Leydig cell isolation and hCG stimulation

Leydig cells were isolated from 8-week-old WT and *Parl*^−/−^ testes using a discontinuous Percoll (Cytiva) density gradient with slight alterations as described before^55^. Both testes of freshly sacrificed mice were washed, decapsulated and added to a collagenase IV solution with 1 mg/mL collagenase IV in medium for 15 min at 37 °C. To end digestion Medium containing fetal calf serum was added and the mix left to settle for a few minutes. The supernatant was filtered through a cell strainer (40 µm) and centrifuged for 5 min at 500 × g. The pellet was resuspended in 1 ml of HBSS and the solution layered on top of a discontinuous Percoll gradient with 60%, 50%, 40% and 30% Percoll in HBSS and centrifuged for 30 min at 800 × *g* at 4 °C. The Percoll fractions were isolated washed and centrifuged for 5 min at 500 × *g*. The cells were resuspended in 500 µL to 1 mL of DMEM + 10% FCS + 100 U/mL penicillin + 100 µg/mL streptomycin, counted using Typan blue (Gibco), and seeded with 1.5 × 10^5^ cells per well on a 96-well plate. The cells were incubated at 5% CO_2_ and 37°C for 2 h, the medium was exchanged and the cells incubated. After 48 h the medium was taken off and the cells were stimulated with human chorionic gonadotropin (hCG) (Ferring) at a concentration of 10 mIU/mL for 2 h. To measure basal testosterone production one well per animal was not stimulated and medium without hCG was added. After 2 h the medium was removed, centrifuged and the supernatant snap frozen. Cells were washed with PBS, harvested using trypsin, centrifuged for 5 min at 500 × *g* and resuspended in PBS. A small droplet of the cell suspension was put on a microscope slide, left to dry and a 3-β-HSD-assay was performed as described by Liang et al.^56^ to assess the purity of Leydig cells. Dried cells were washed with PBS for 1 min, then the 3-β-HSD-assay-solution (0.5 mg Nitro blue Tetrazolium Chloride (NBT), 0.3 mg DHEA, 0.3 mL DMSO, 5 mg β-NAD, 4.75 mL PBS) was added and incubated for 30 min at 37 °C. The microscope slide was rinsed with PBS and left to dry. Purity was >85% with only minor infiltration of germ cells.

### Testosterone measurement in cell culture medium

The testosterone level in the cell culture medium of the isolated Leydig cells was measured using a Testosterone ELISA Kit (Cayman, range: 3.9–500 pg/mL, sensitivity: ~6 pg/mL) following the manufacturer’s instructions. Samples were diluted at 1:50, 1:100 and 1:200 and tested together with standards in duplicates with the reaction mix on the pre-coated 96-well plate. After 2 h of incubation, wells were washed and Ellman´s reagent was added. Absorption was measured at 415 nm after 75 min incubation and testosterone levels were calculated using the standard curve.

### Statistics and reproducibility

Statistical analyses were performed using GraphPad PRISM 9.0.0. Data with *n* ≤ 5 were statistically analyzed with a Mann–Whitney *U* Test or a Kruskal–Wallis Test with post hoc pairwise comparison (for *p* ≤ 0.05) and visualized in boxplots (center line: median, box limits: upper and lower quartiles, whiskers: 1.5 × interquartile range), with the significance level being indicated with asterisks (*p ≤ 0.05; **p ≤ 0.01; ***p ≤ 0.001; ****p ≤ 0.0001). Data with *n* > 5 were tested for normality using Kolmogorov–Smirnov tests and in case of normality (*p* > 0.05), data were subsequently analyzed using an unpaired *t* test or ANOVA with *post hoc* pairwise comparison (Tukey’s multiple comparisons test) for *p* ≤ 0.05. Data were visualized in bar charts, with error bars indicating standard deviation and asterisks indicating significance level (*p ≤ 0.05; **p ≤ 0.01; ***p ≤ 0.001; ****p ≤ 0.0001).

For the measurement of testis weight, the average weight of both testes per animal was calculated, and for the analyses of tubule diameter, the average diameter of 10 seminiferous tubules of eight mice for each genotype was calculated.

To analyze the measurements of ATP and LH concentration, the measured concentration was considered in relation to the overall protein concentration of the homogenized samples (in the case of ATP concentration of the original sample before deproteinization) by dividing the measured ATP/LH level through the measured protein concentration (µg/mL).

The results of the RT-qPCRs were analyzed using the 2^-ΔΔCT^ method^57^. ΔCT values were calculated by subtracting the CT value of the reference gene (*Gapdh*) from the CT value of the target gene. The ΔΔCT value was then calculated by subtracting the median ΔCT value of the WT from the ΔCT value of each WT and *Parl*^−/−^ sample.

To analyze the protein expression of the isolated mitochondria using Western blot, the density of bands was measured using ImageJ. Relative protein expression was calculated by dividing the values of the target protein by the value of a reference protein (HSP60, aconitase 2 (ACO2)). To calculate the expression relative to the WT control, all values were divided by the median of the WT control group.

### Reporting summary

Further information on research design is available in the Nature Portfolio Reporting Summary linked to this article.

### Supplementary information

Supplementary information

Description of additional supplementary files

Supplementary data 1

Reporting Summary - Flat

## Data Availability

All data supporting the findings of this study are available within the paper and its Supplementary Information. Uncropped and unedited images of all Western blots are provided in the Supplementary Information (Figs. S6–S14) and numerical source data to all graphs are provided in the Supplementary Data 1 file.
