# Supplementary material for: The mitochondrial protease PARL is required for spermatogenesis
Source: Commun Biol. 2024 Jan 5;7:44. doi: 10.1038/s42003-023-05703-3 (PMC10770312; doi:10.1038/s42003-023-05703-3)
Supplement: Supplementary file 1 — Supplementary information [file 42003_2023_5703_MOESM1_ESM.pdf]

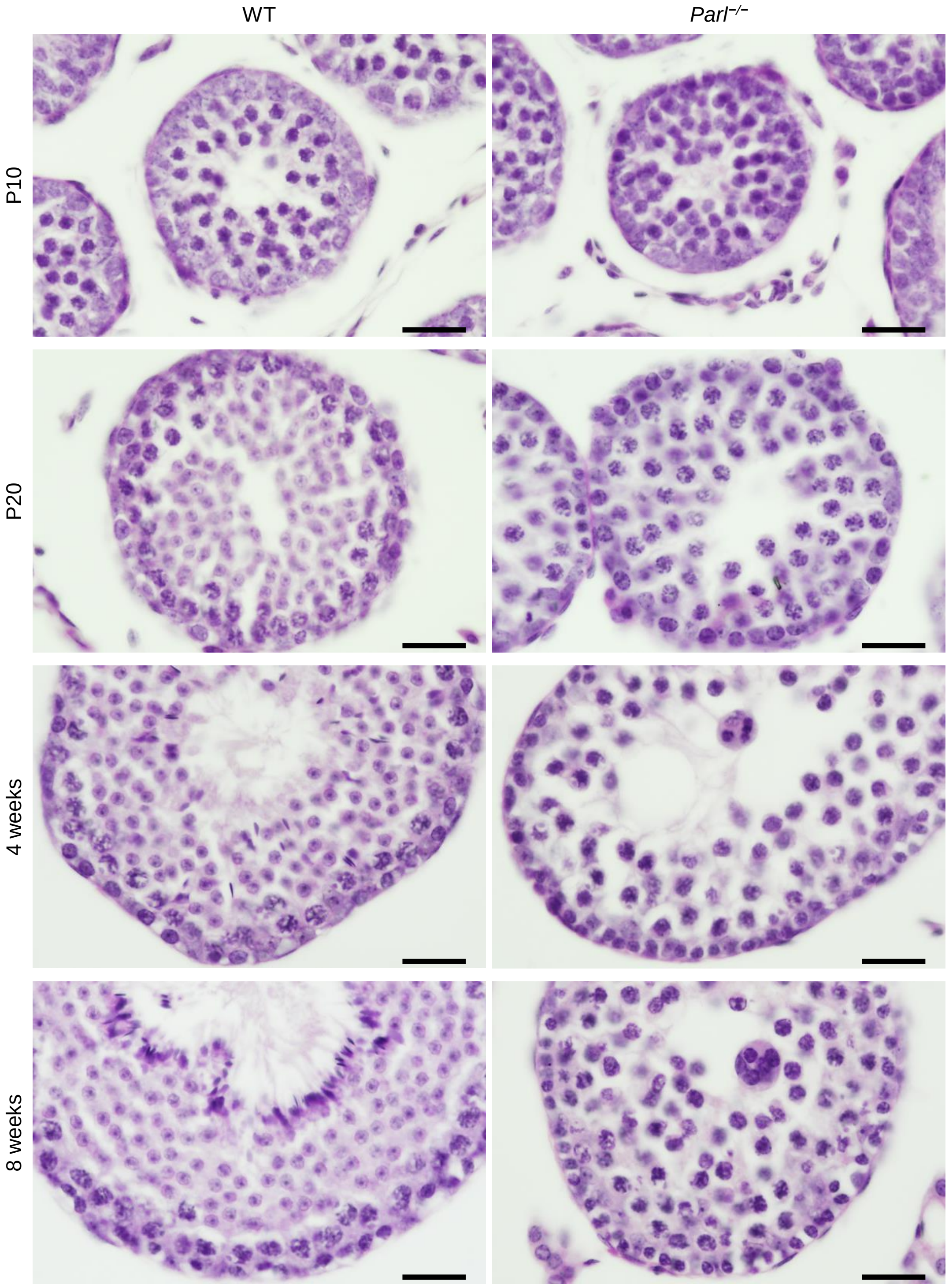

Fig. S1: Histological overview of the effect of PARL deficiency on spermatogenesis.

PAS staining of seminiferous tubules at the age of 10 days, 20 days, 4 weeks and 8 weeks of WT and *Parl*$^{-/-}$ mice (scale bar: 25 µm).

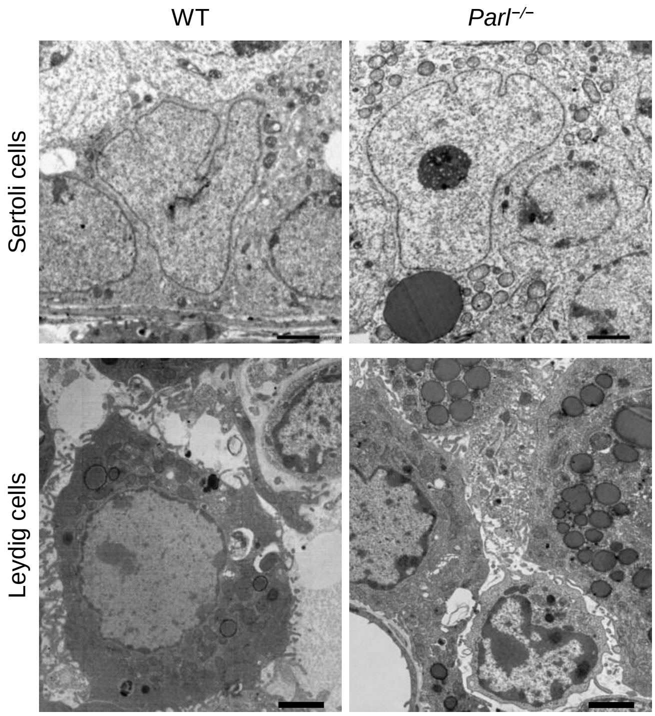

Fig. S2: Examples of Sertoli and Leydig cells

TEM pictures of Sertoli cells and Leydig cells of 8-week-old WT and *Parl*$^{-/-}$ mice (scale bars: 2 µm).

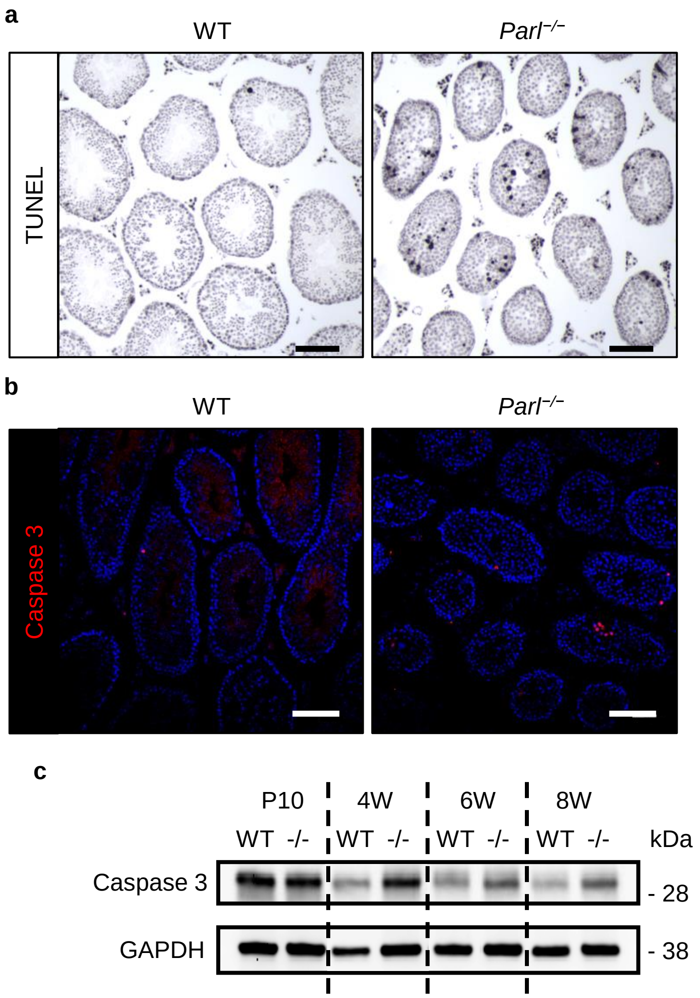

Fig. S3: PARL deficiency leads to an increased number of apoptotic germ cells.

a TUNEL assay of *Parl*$^{-/-}$ testes in comparison to WT litter mates at the age of 8 weeks. The black-stained cells indicate apoptotic cells (scale bars; left: 100 µm, right: 50 µm). **b** Immunohistological staining of WT and *Parl*$^{-/-}$ testes of 8-week-old mice with caspase 3, marking apoptotic cells (scale bars; left: 100 µm, right: 50 µm). (c) Western blot of testes of WT and *Parl*$^{-/-}$ mice at the age of 10 days, 4 weeks, 6 weeks, and 8 weeks for caspase 3. GAPDH was used as an internal loading control.

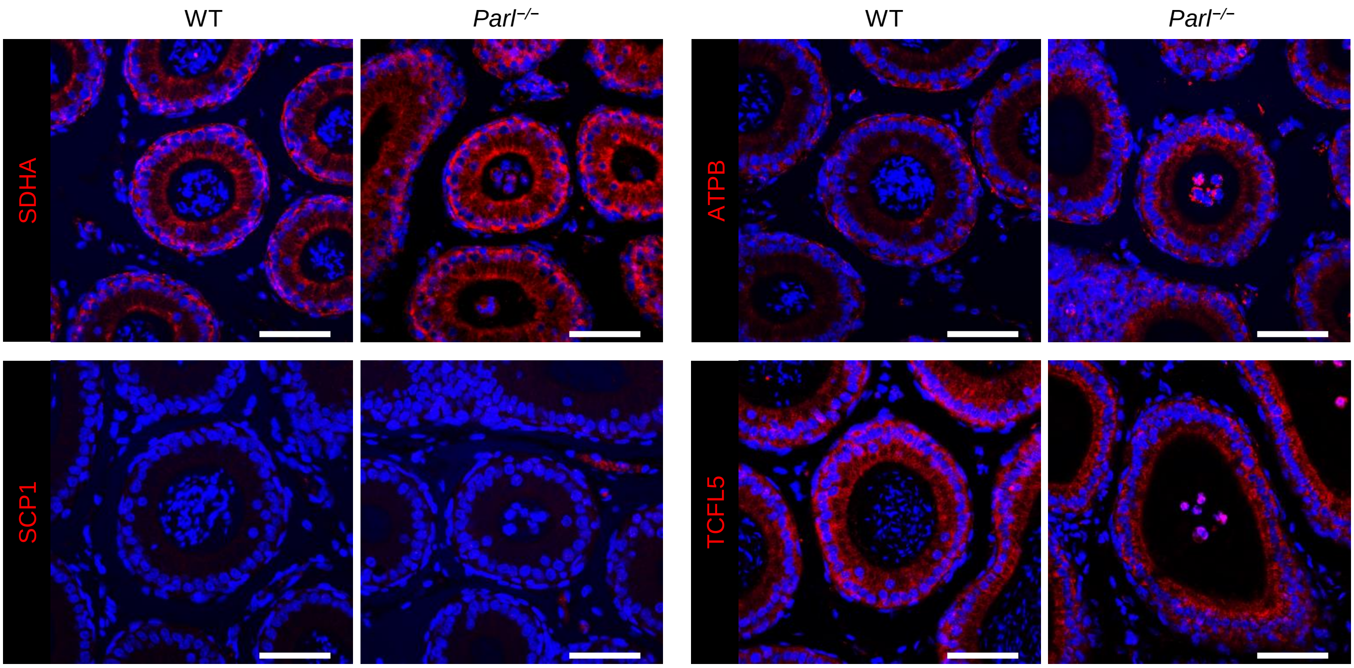

Fig. S4: Diplotene spermatocytes were found in the epididymides of PARL-deficient mice.

Immunohistological fluorescence staining of WT and *Parl*−/− epididymides at the age of 8 weeks with SDHA and ATPB (mitochondrion markers), SCP1 (zygotene / early diplotene spermatocytes), and TCFL5 (diplotene spermatocytes) (scale bars: 50 µm).

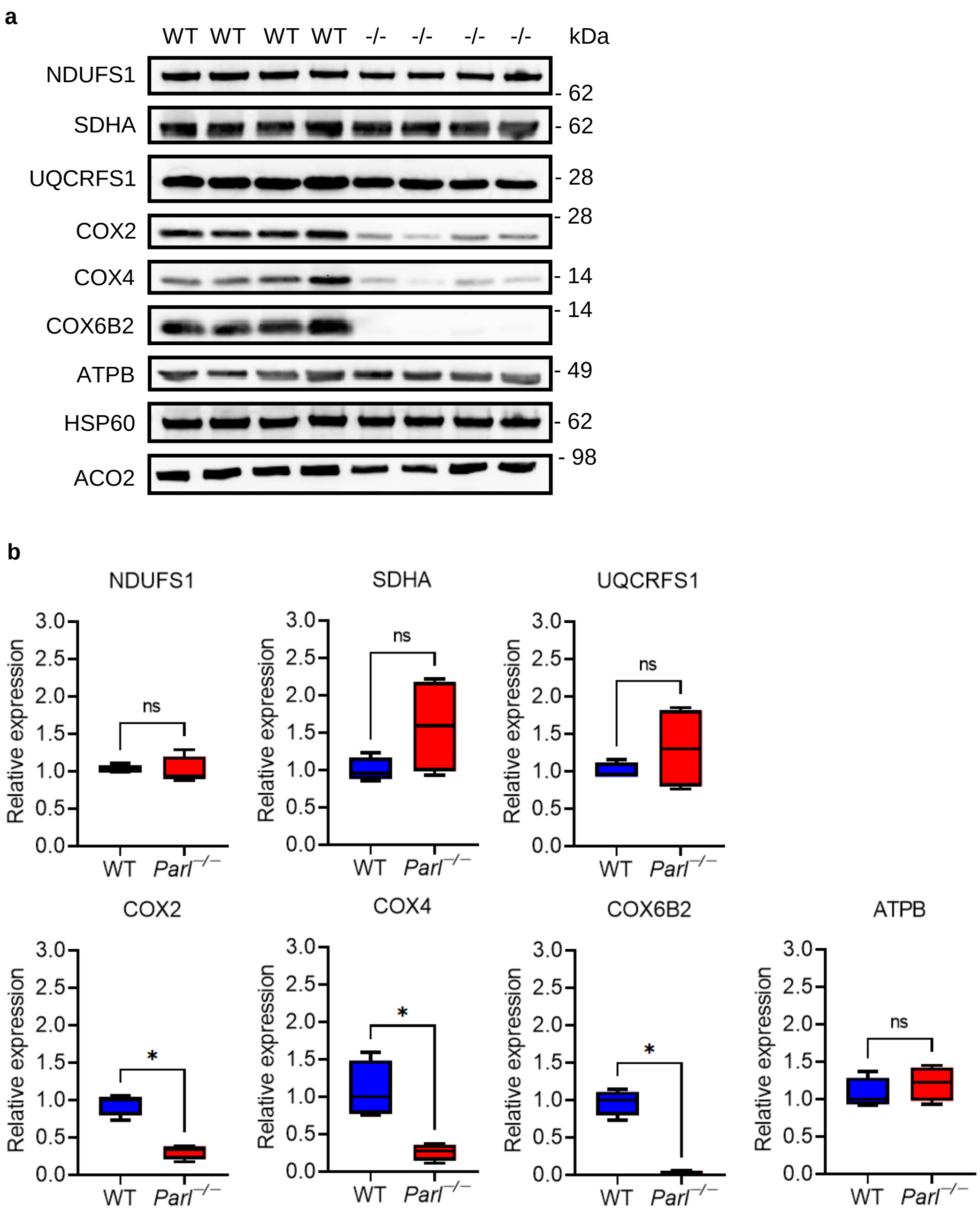

Fig. S5: Expression of proteins of respiratory chain complex I-V

a Western blots of proteins of respiratory chain complexes of isolated mitochondria of 8-week-old WT and *Parl*$^{-/-}$ mice. HSP60 or ACO2 was used as a loading control (for the membrane with SDHA and UQCRFS1 ACO2 was used as loading control because SDHA are too close in molecular weight). **b** Relative expression of respiratory chain complex proteins based on the western blots. Graphical density values of the target protein were divided by the values of the loading control (HSP60 or ACO2) and then divided by the median value of the WT control group. Statistics: Mann–Whitney U test: NDUFS1: $p = 0.3429$, SDHA: $p = 0.3429$, UQCRFS1: $p > 0.9999$, COX2: $p = 0.0286$, COX4: $p = 0.0286$, COX6B2: $p = 0.0286$, ATPB: $p = 0.4857$.

Table S1: List of antibodies used in this study.

Protein	Company	Catalog no.	Concentrations IHC	Concentrations WB
ABP	Abcam	ab126617	-	1:1000
ACO2	Abcam	Ab129069	1:100	1:25,000
ACRV1	Proteintech	14040-1-AP	1:100	1:2000
ATPB	Proteintech	17247-1-AP	1:100	1:4000
BHMT	Novous Bio	NBP1-00161	1:50	1:2000
Caspase3	Cellsignaling	9662	1:50	1:500
Claudin11	Abcam	Ab53041	1:100	-
COQ4	Proteintech	16654-1-AP	-	1:1000
COX2 (MTCO2)	Proteintech	55070-1-AP	1:50	1:2500
COX4	Proteintech	11242-1-AP	1:100	1:5000
COX6B2	Proteintech	11437-1-AP	1:100	1:500
CYP11A1	Proteintech	13363-1-AP	1:100	1:1000
Cyp17A1	Proteintech	14447-1-AP	1:100	1:2000
DDX4	Abcam	Ab13840	1:100	1:1000
Diablo(SMAC)	Proteintech	10434-1-AP	1:50	1:2000
GAPDH	HyTest	HyTest 5G4	-	1:10,000
HSD17B3	Biorbyt	BYT-ORB5476	1:50	1:250
HSD17B12	Invitrogen	PA5-69454	-	1:1000
HSP60	Proteintech	66041-1-lg	-	1:25,000
INHBA	Proteintech	17524-1-AP	-	1:500
MFN1	Proteintech	13798-1-AP	1:100	1:10,000
MFN2	Proteintech	12186-1-AP	1:100	1:10,000
NDUFS1	Proteintech	12444-1-AP	1:50	1:2000
PCNA	Abcam	Ab18197	1:100	1:1000
PGAM5	Abcam	Ab126534	-	1:500
SCP1	Novus Bio	NB300-229SS	1:200	1:1000
SDHA	Abcam	Ab14715	1:100	1:1000
SQRDL	Novus Bio	NBP1-84510	-	1:1000
StAR	Proteintech	12225-1-AP	1:100	1:1000
STARD7	Proteintech	15689-1-AP	-	1:500
SULT1E1	Proteintech	12522-1-AP	1:50	1:500
TCFL5	Proteintech	29404-1-AP	1:50	1:1000
Transferrin	Proteintech	17435-1-AP	-	1:1000
TTC19	SigmaAldrich	HPA052380	-	1:1000
UQCRFS1	Proteintech	18443-1-AP	1:50	1:4000
Vimentin	Abcam	Ab92547	1:100	1:1000

Fig. S6: Uncropped images of Western Blots Fig. 2c

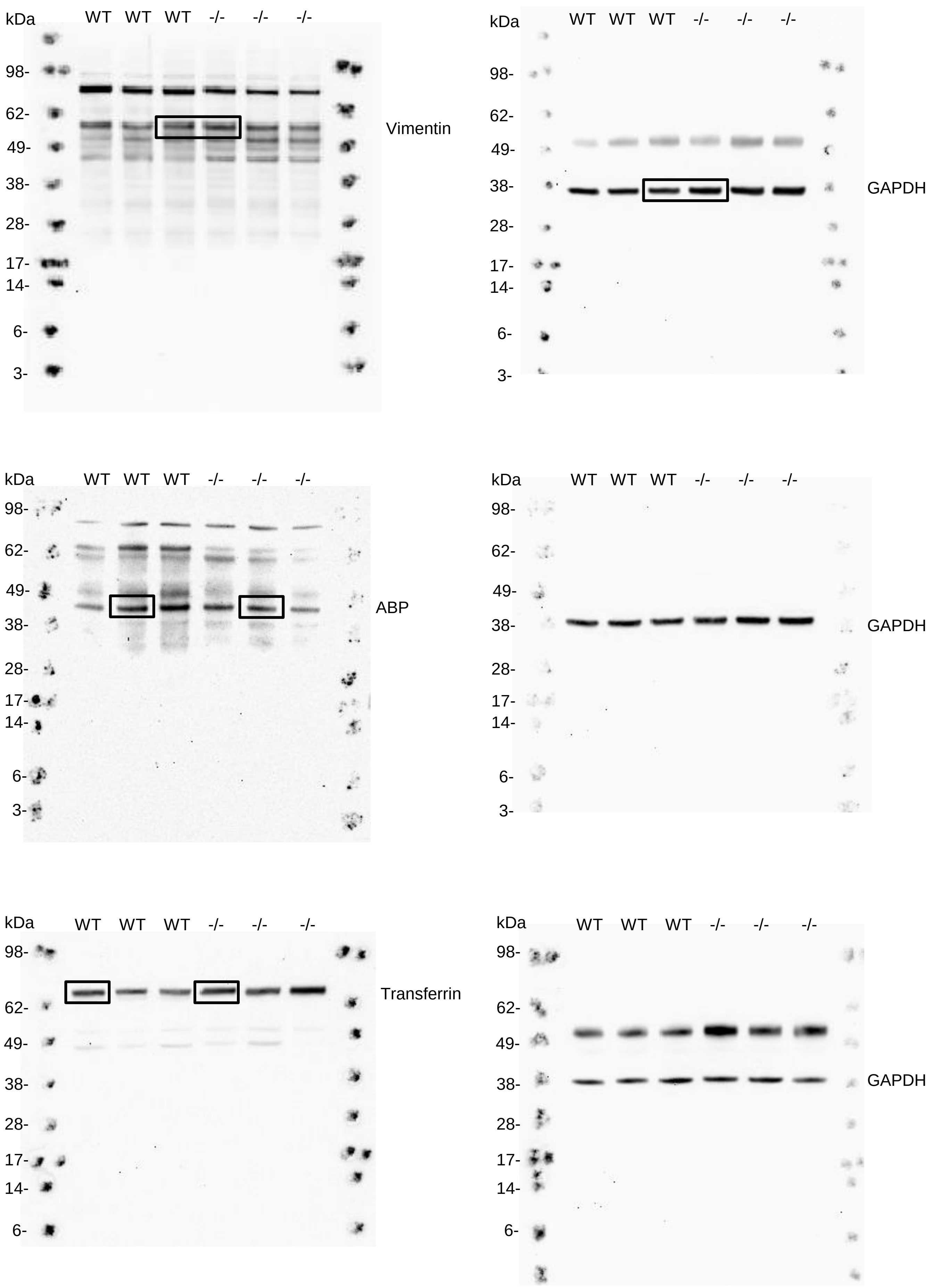

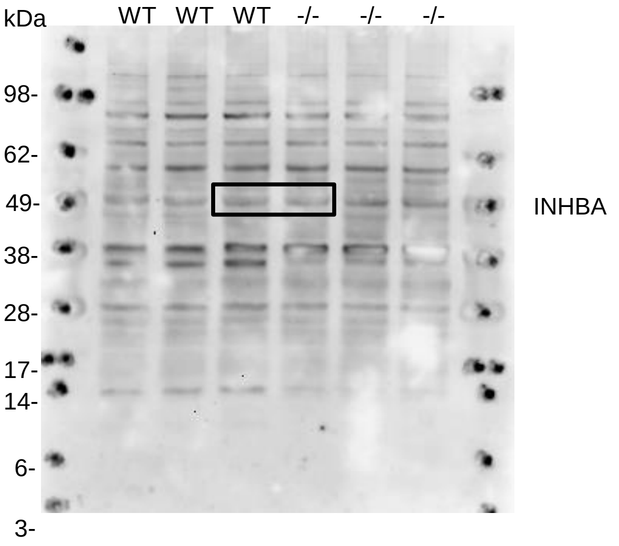
kDa
WT WT WT -/- -/- -/-
98-
62-
49-
38-
28-
17-
14-
6-
3-
INHBA

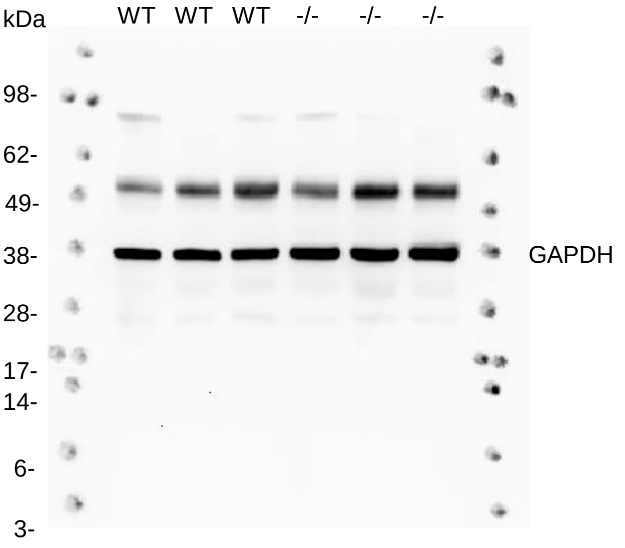
kDa
WT WT WT -/- -/- -/-
98-
62-
49-
38-
28-
17-
14-
6-
3-
GAPDH

Fig. S7: Uncropped images of Western Blots Fig. 2d

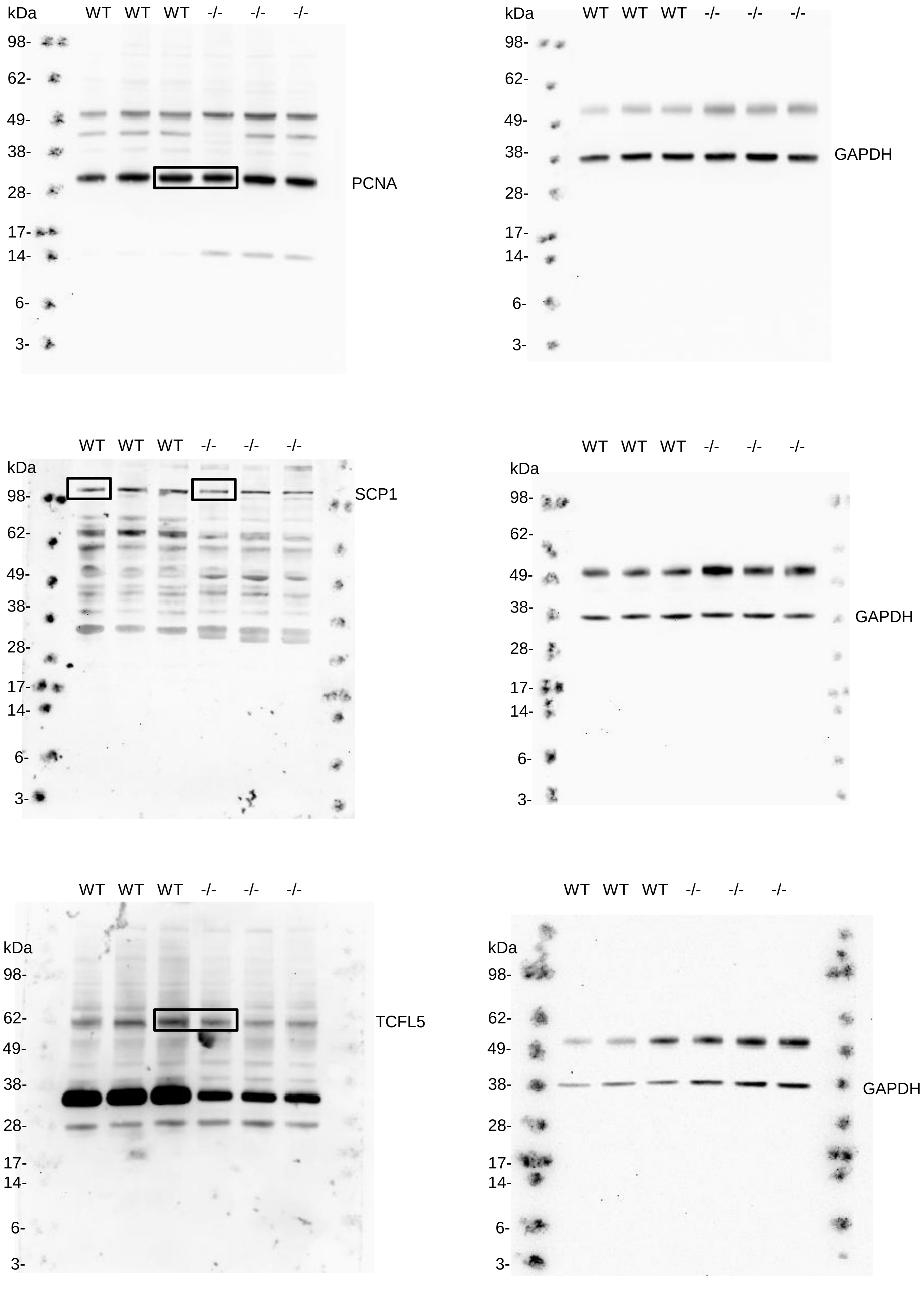

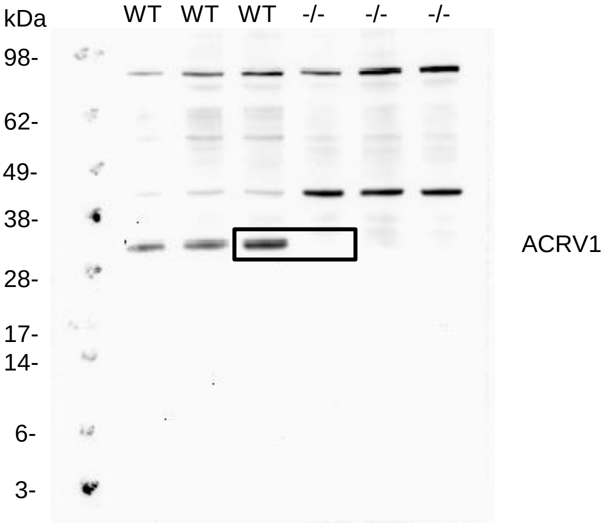
kDa
WT WT WT -/- -/- -/-
98-
62-
49-
38-
28-
17-
14-
6-
3-
ACRV1

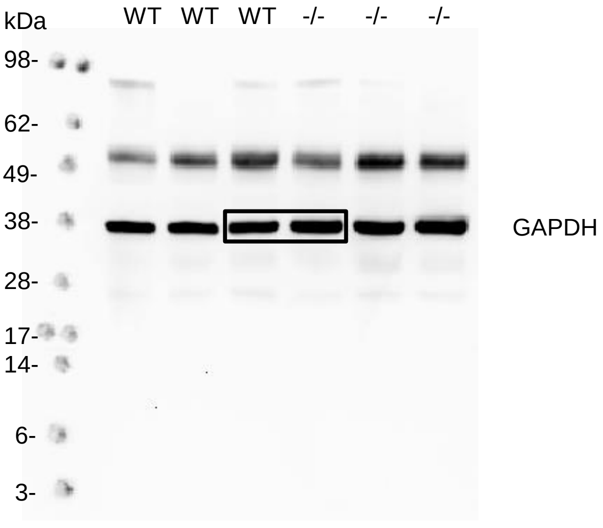
kDa
WT WT WT -/- -/- -/-
98-
62-
49-
38-
28-
17-
14-
6-
3-
GAPDH

Fig. S8: Uncropped images of Western Blots Fig. 4a

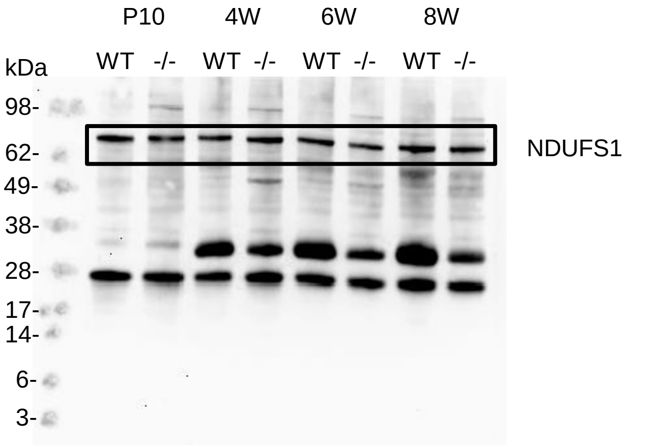

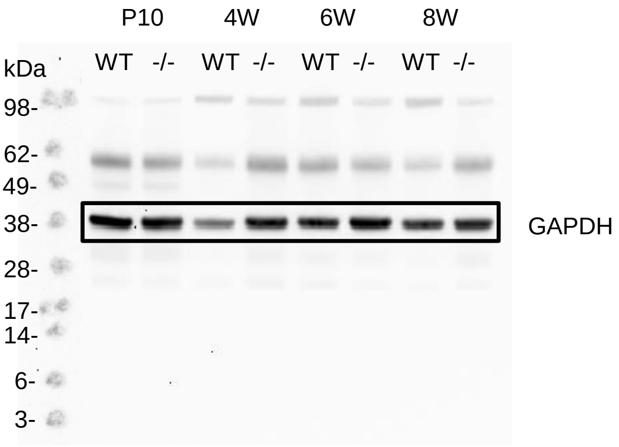

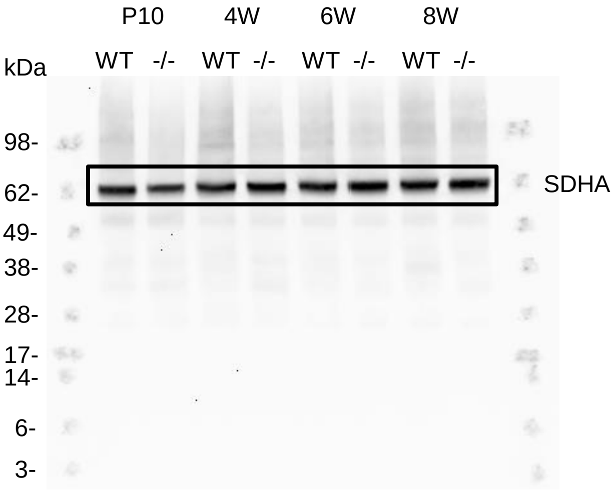

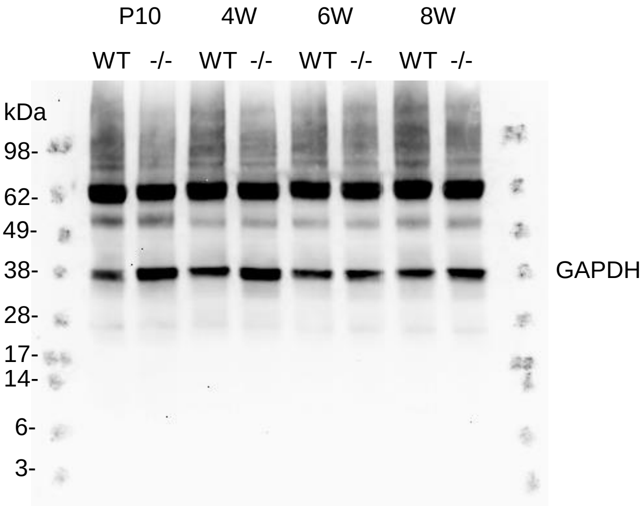

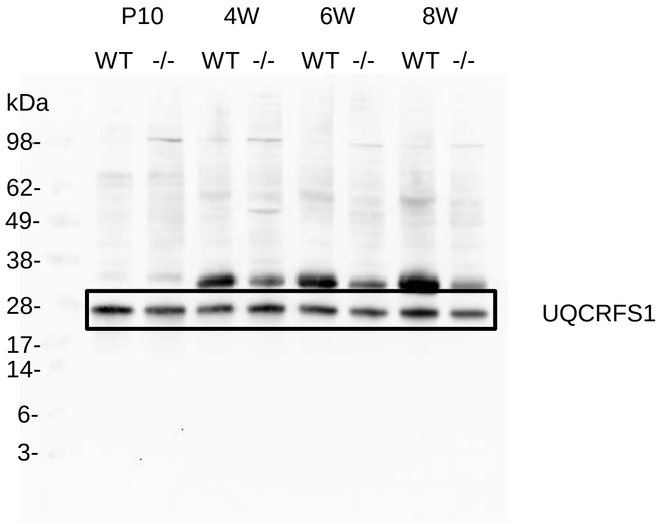

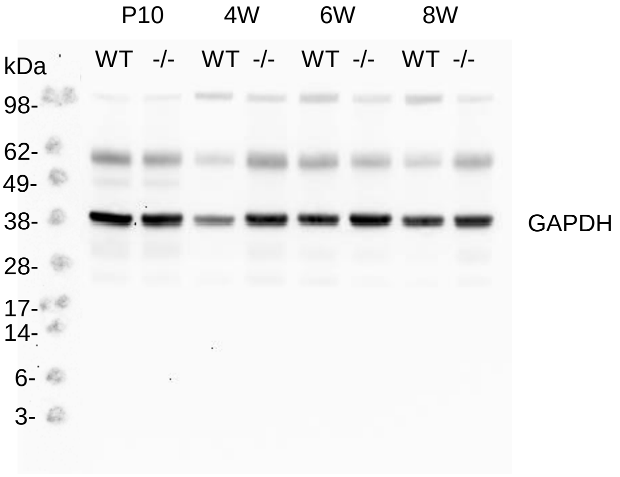

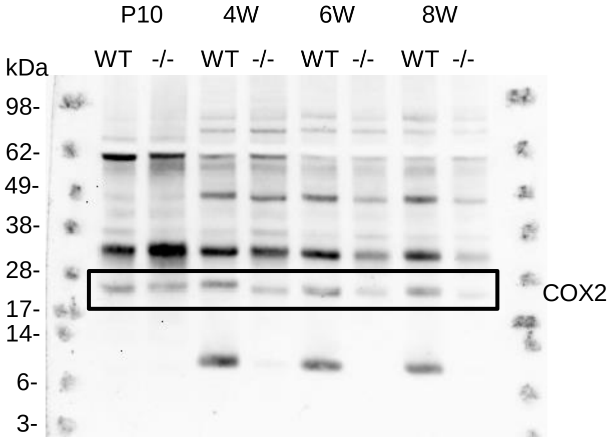

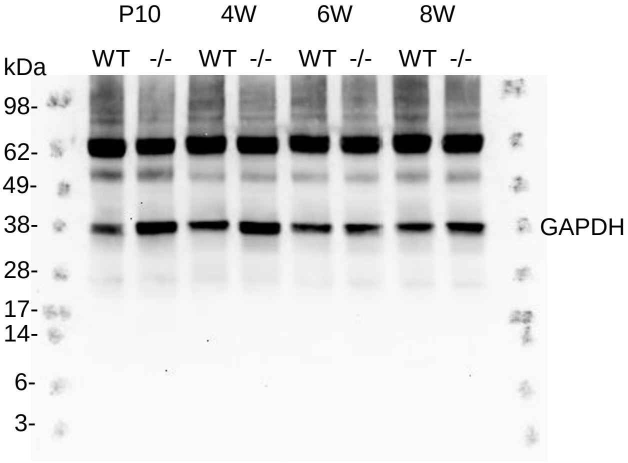

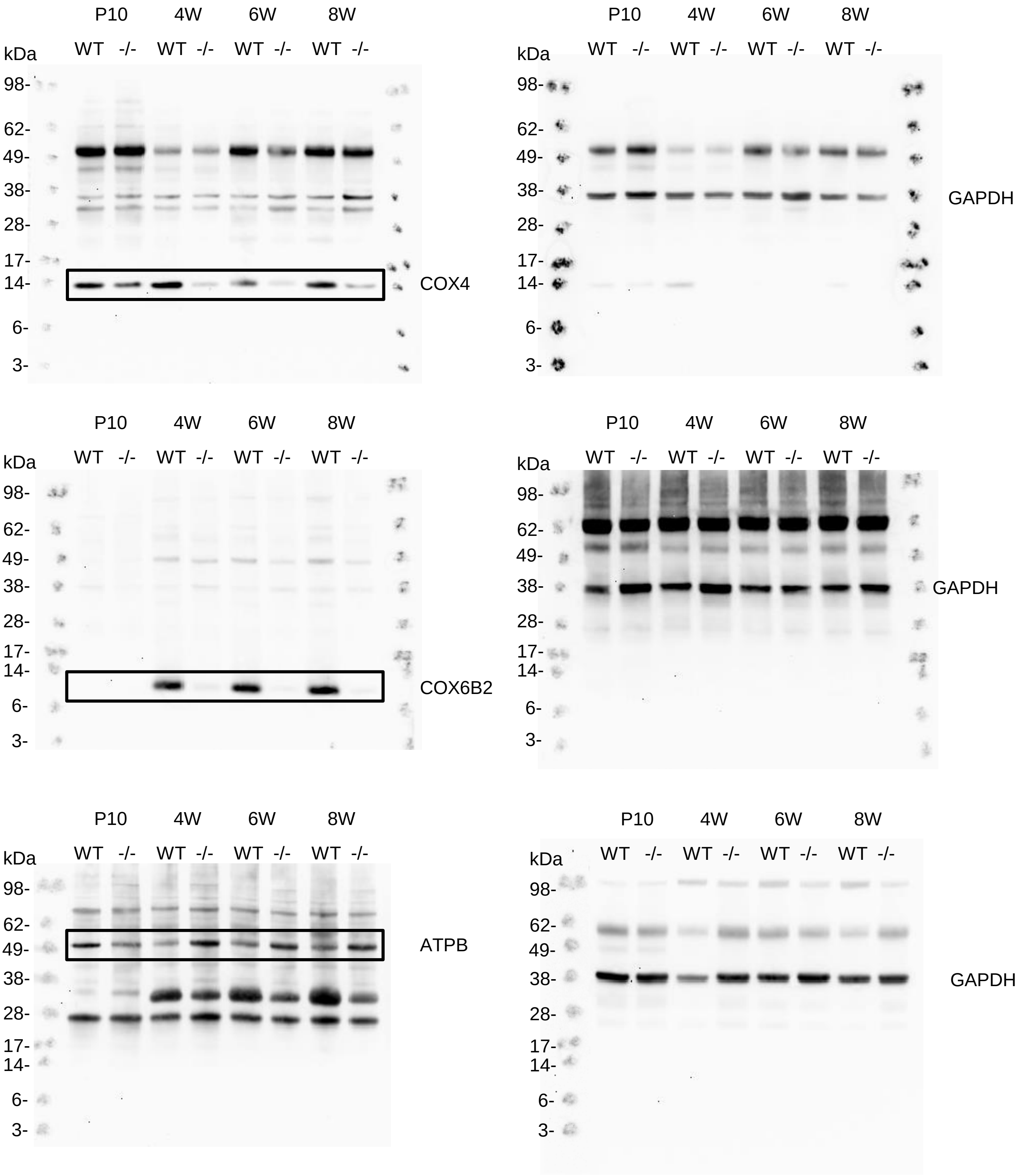
P10 4W 6W 8W
WT -/- WT -/- WT -/- WT -/-
kDa
98-
62-
49-
38-
28-
17-
14-
6-
3-
COX4
GAPDH
COX6B2
GAPDH
ATPB
GAPDH

Fig. S9: Uncropped images of Western Blots Fig. 4d

Brain stem

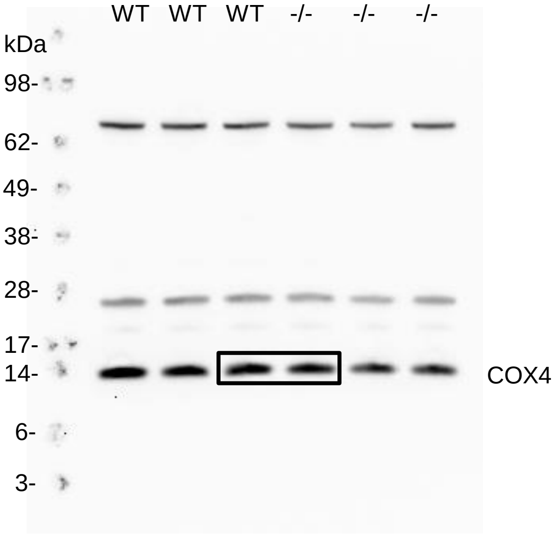

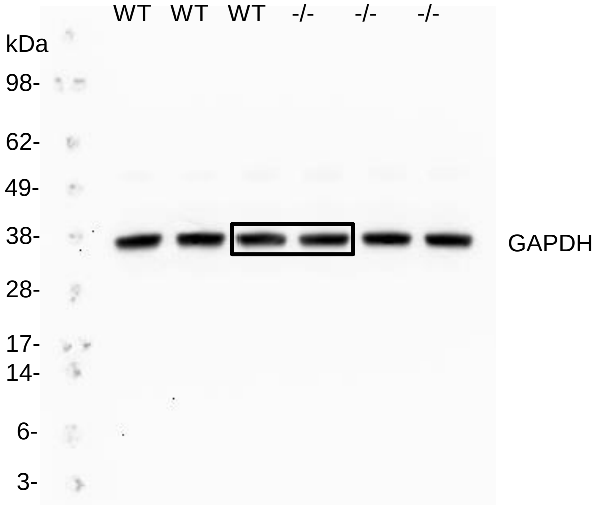

Diencephalon

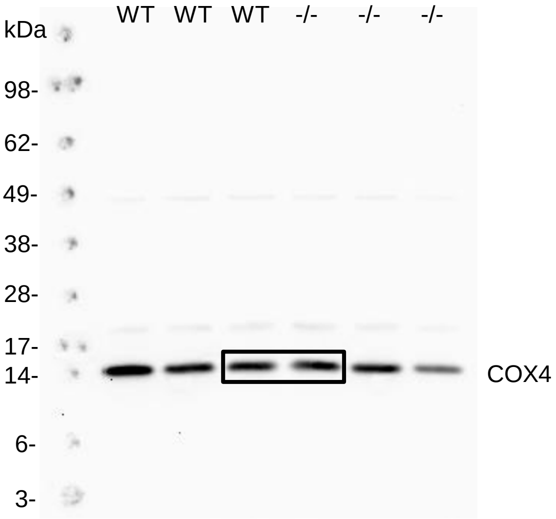

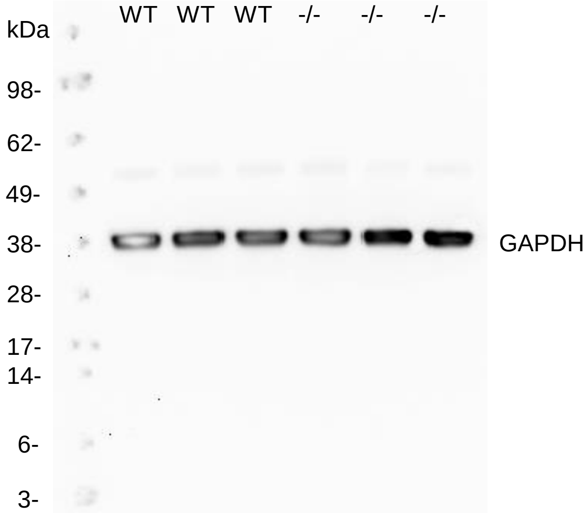

Quadriceps

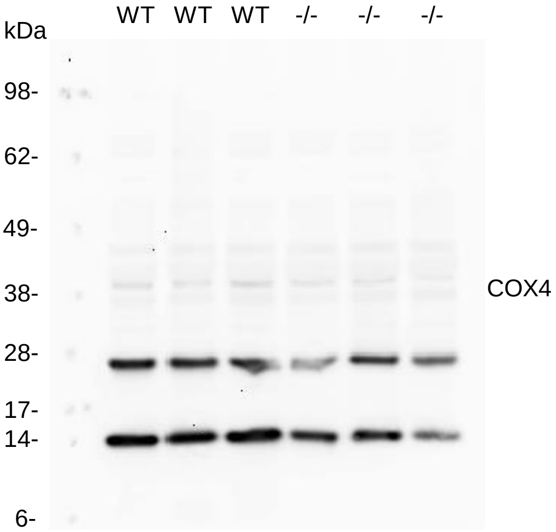

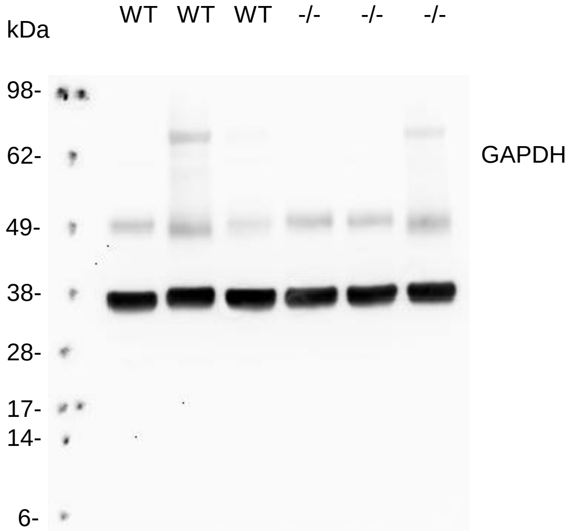

Spleen

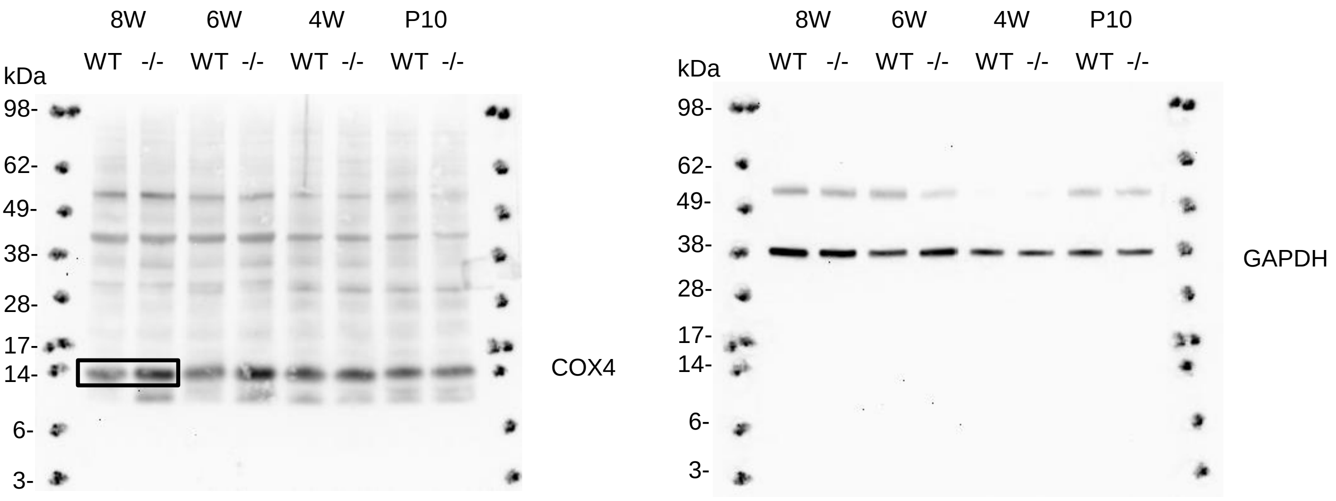

Thymus

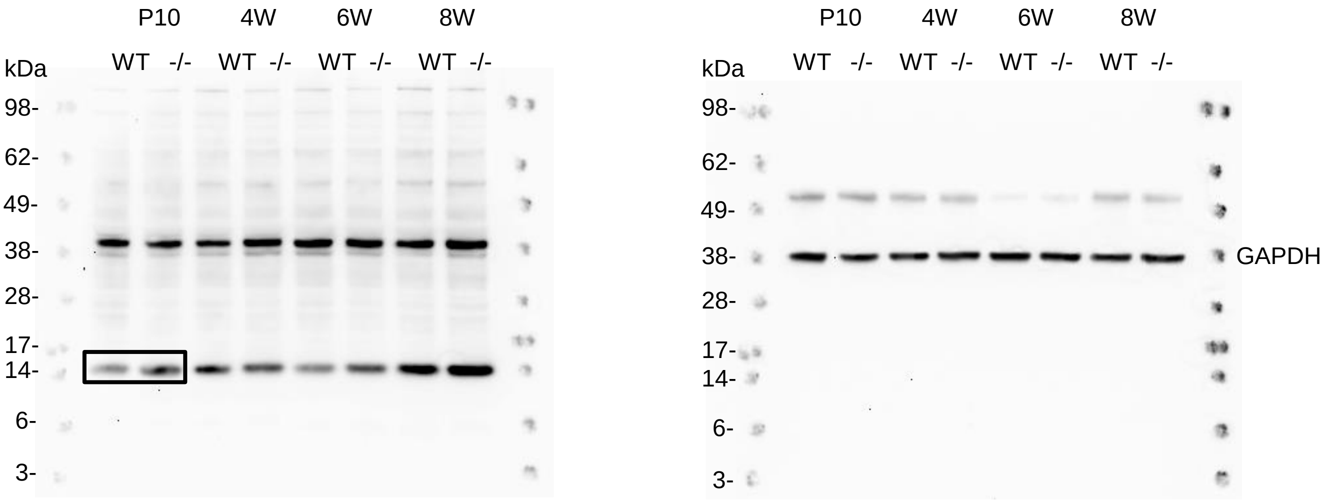

Ovary

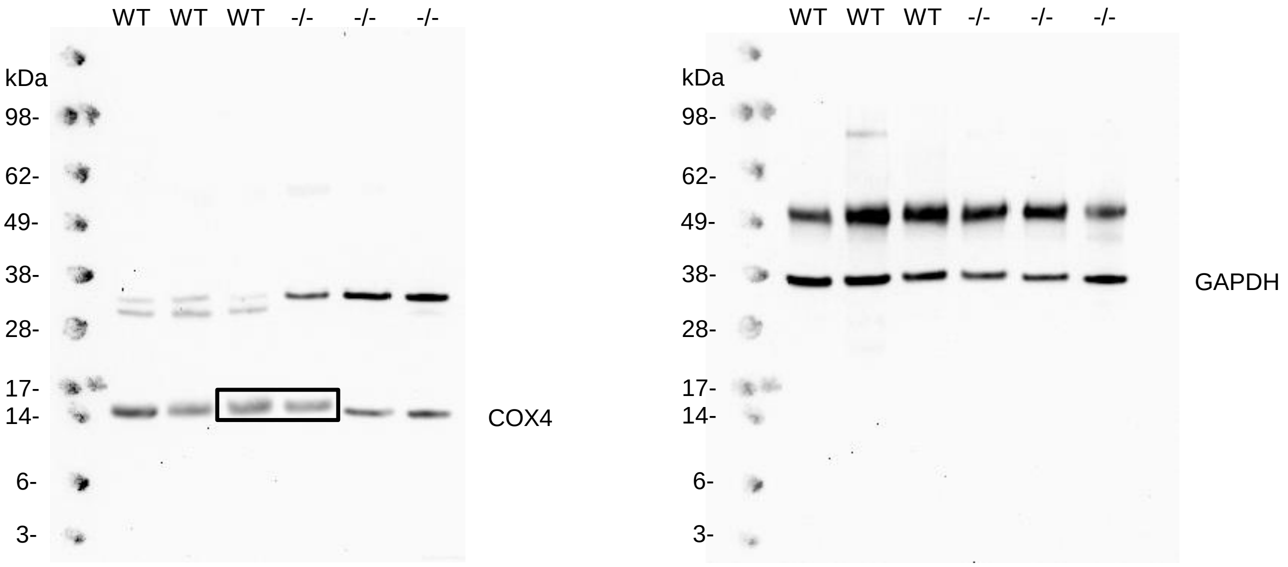

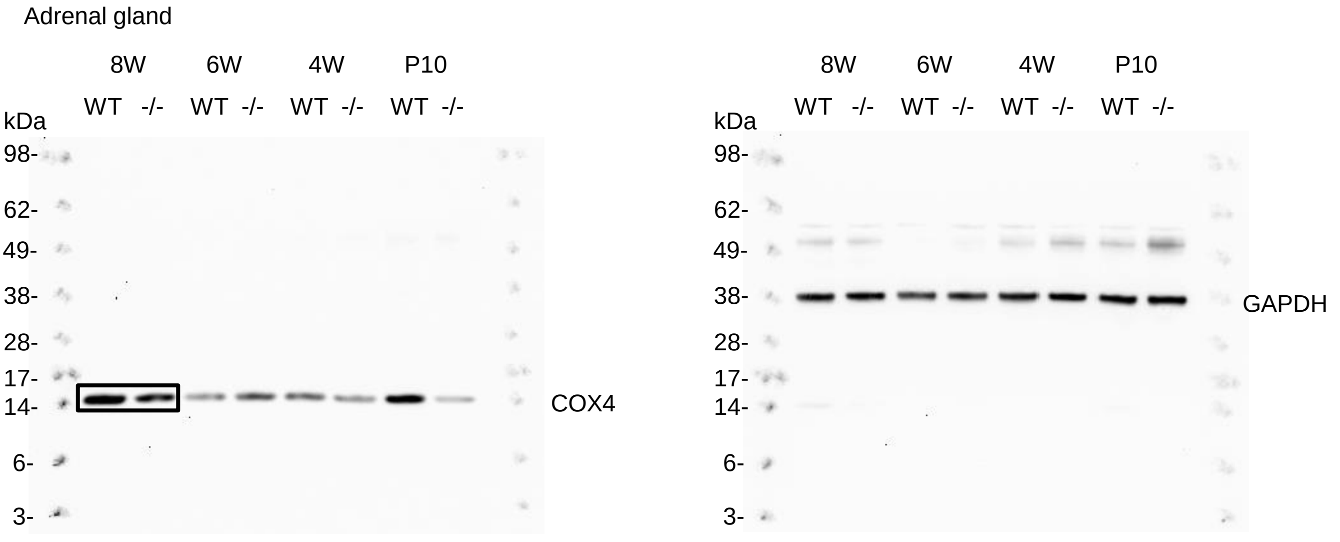

Adrenal gland
8W
6W
4W
P10
WT -/- WT -/- WT -/- WT -/-
kDa
98-
62-
49-
38-
28-
17-
14-
6-
3-
COX4
GAPDH

Fig. S10: Uncropped images of Western Blots Fig. 4e

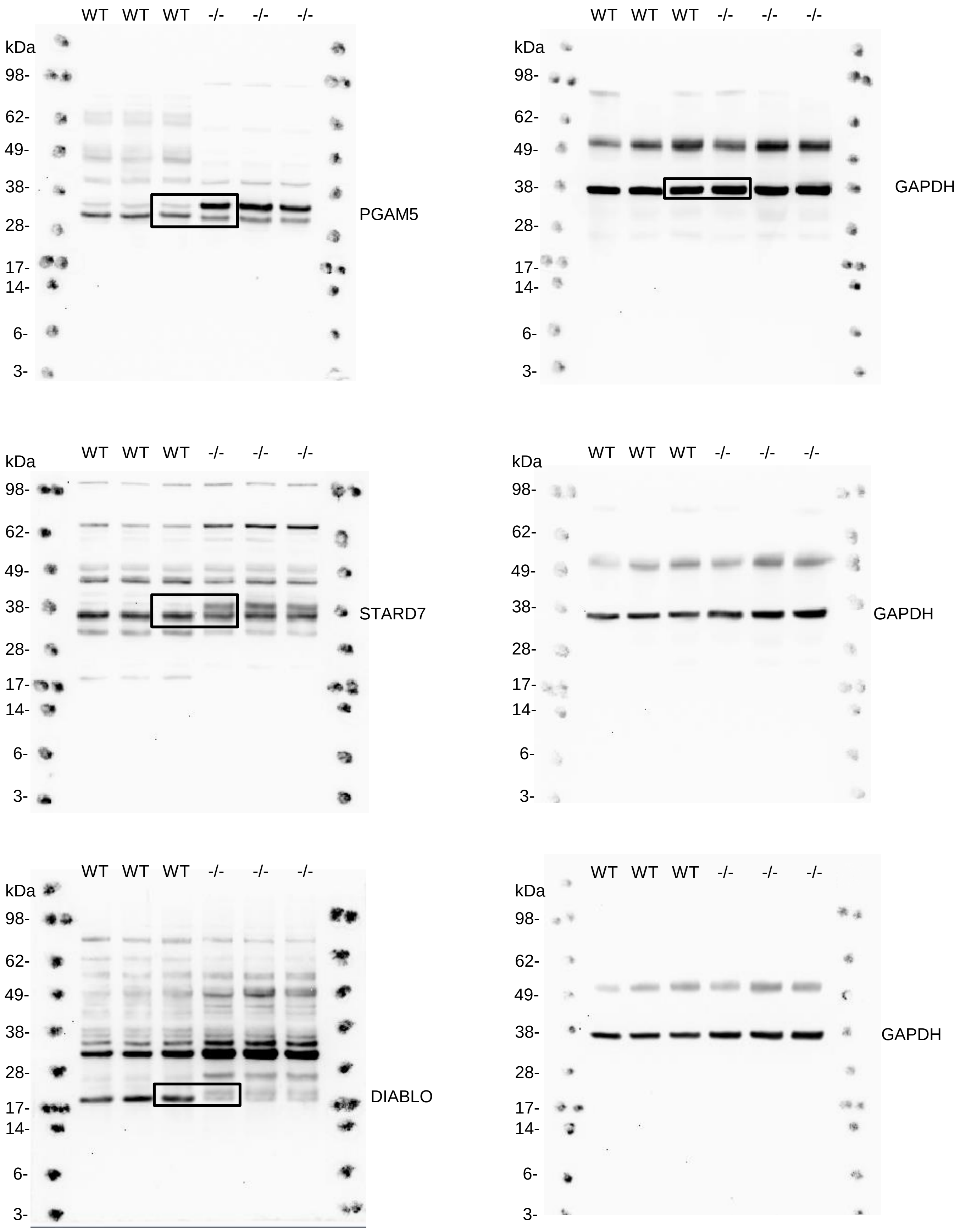

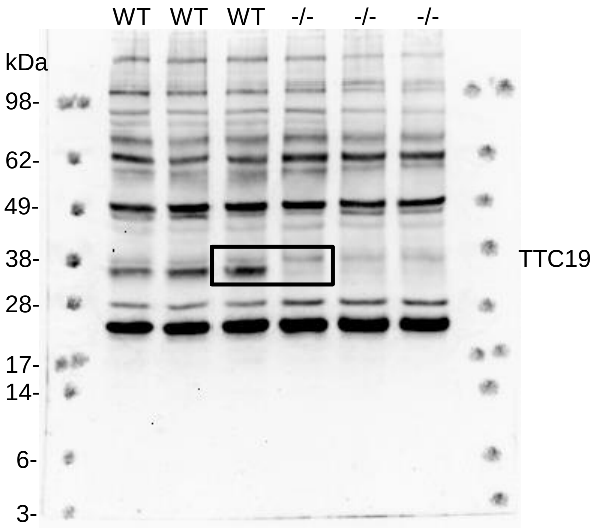
WT WT WT -/- -/- -/-
kDa
98-
62-
49-
38-
28-
17-
14-
6-
3-
TTC19

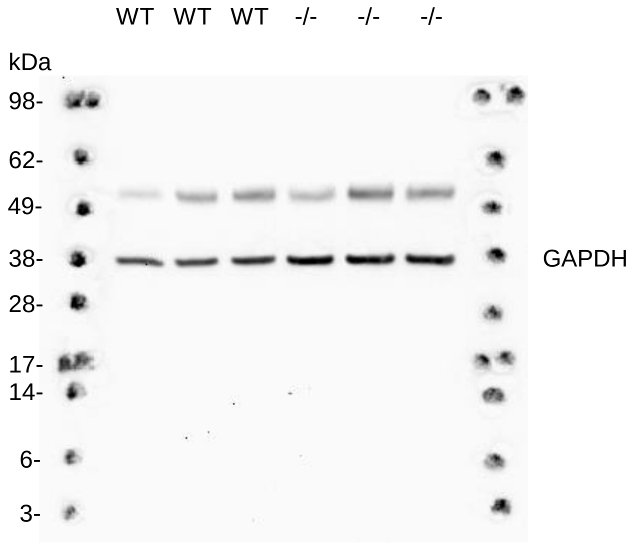
WT WT WT -/- -/- -/-
kDa
98-
62-
49-
38-
28-
17-
14-
6-
3-
GAPDH

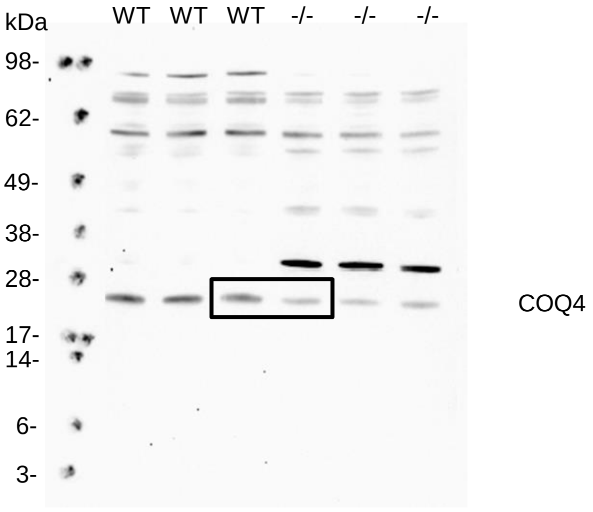
WT WT WT -/- -/- -/-
kDa
98-
62-
49-
38-
28-
17-
14-
6-
3-
COQ4

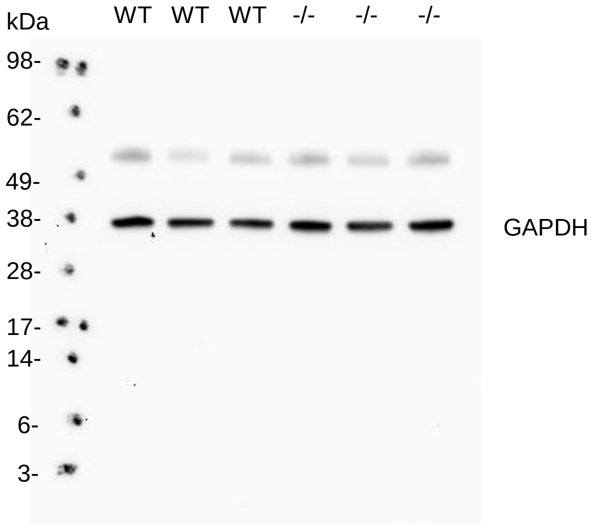
WT WT WT -/- -/- -/-
kDa
98-
62-
49-
38-
28-
17-
14-
6-
3-
GAPDH

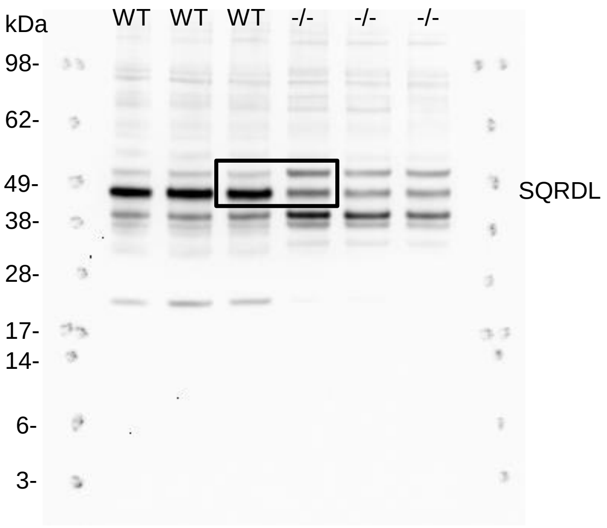
WT WT WT -/- -/- -/-
kDa
98-
62-
49-
38-
28-
17-
14-
6-
3-
SQRDL

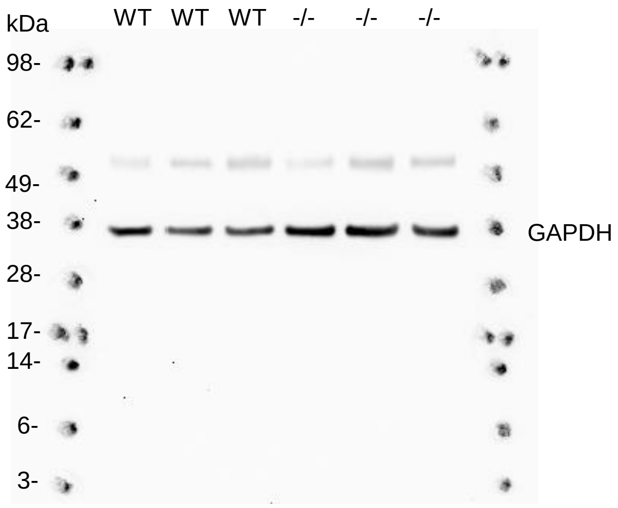
WT WT WT -/- -/- -/-
kDa
98-
62-
49-
38-
28-
17-
14-
6-
3-
GAPDH

Fig. S11: Uncropped images of Western Blots Fig. 7a

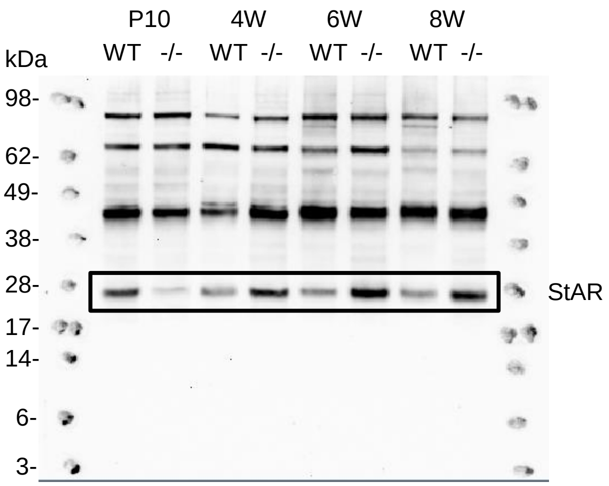

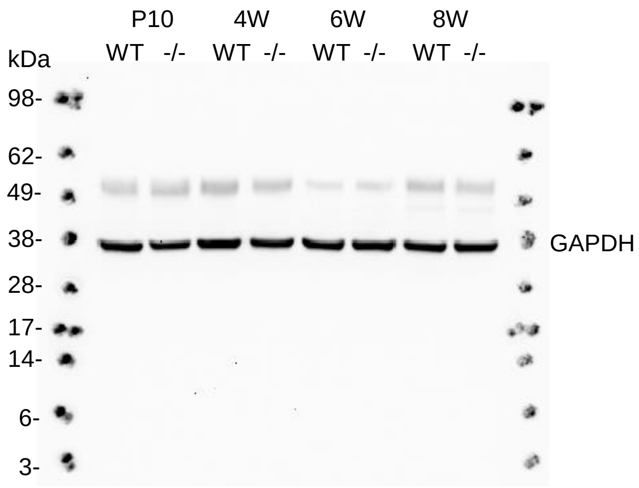

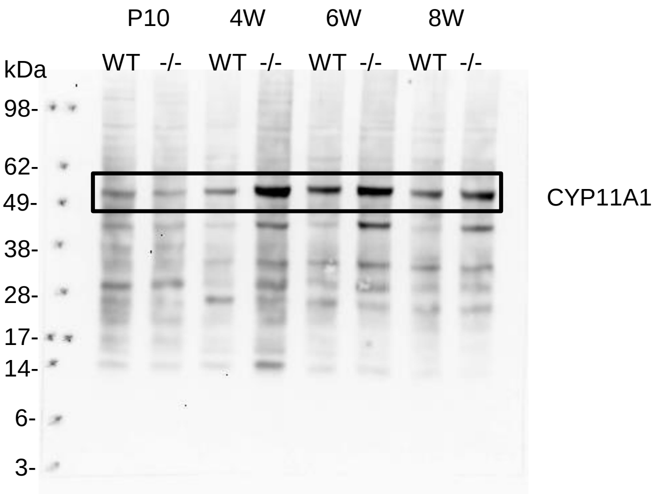

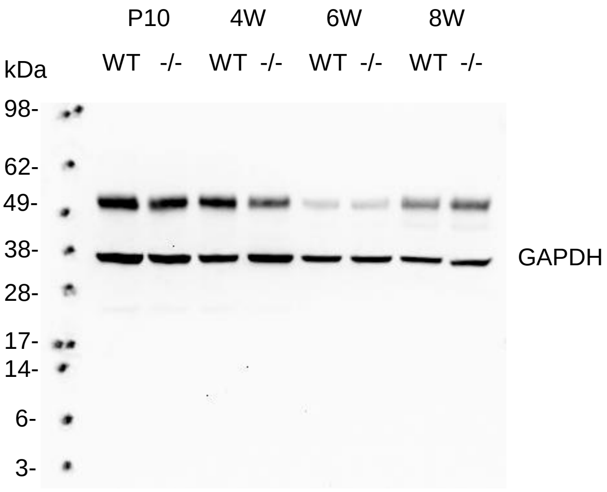

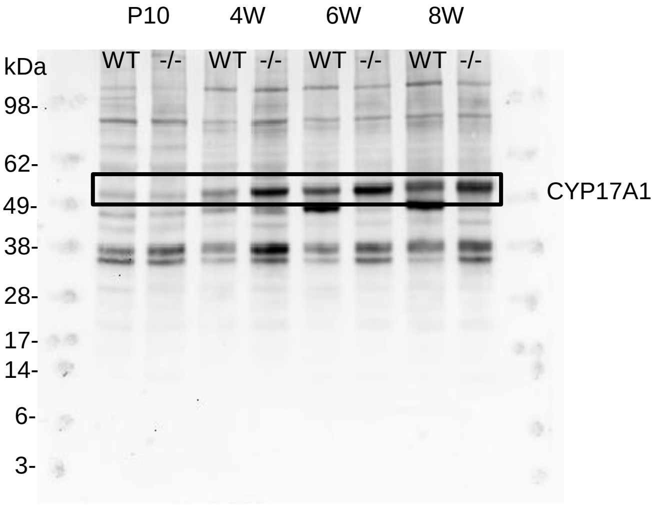

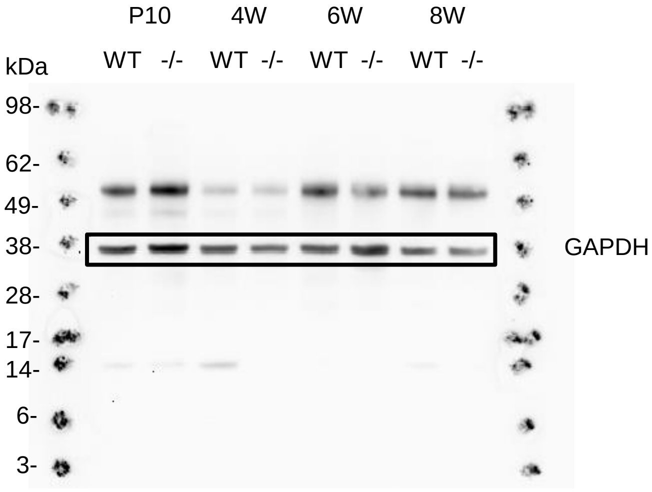

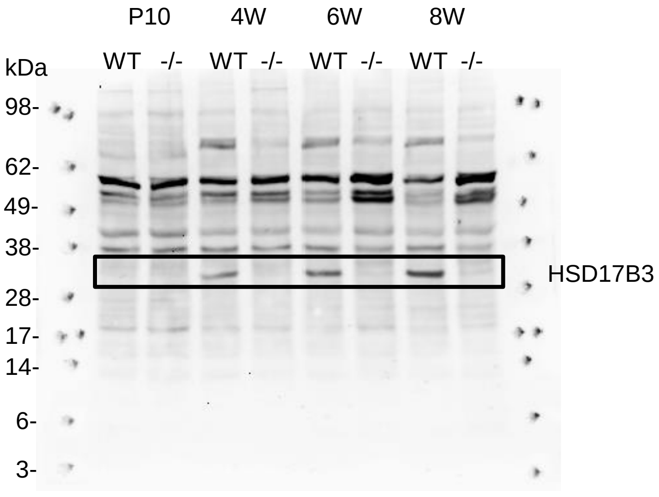
P10 4W 6W 8W
WT -/- WT -/- WT -/- WT -/-
kDa
98-
62-
49-
38-
28-
17-
14-
6-
3-
HSD17B3

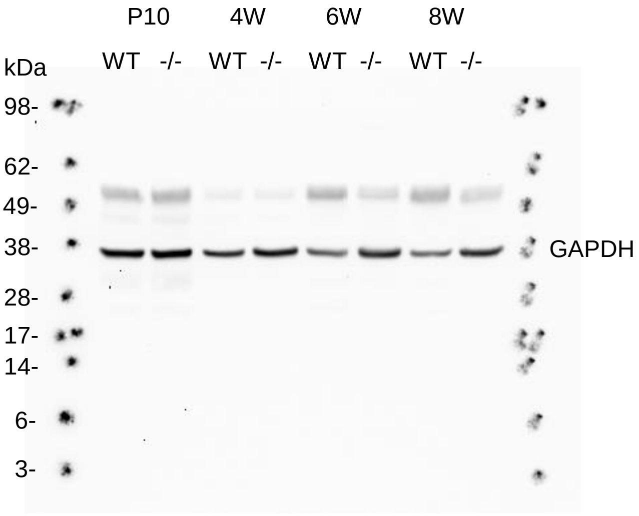
P10 4W 6W 8W
WT -/- WT -/- WT -/- WT -/-
kDa
98-
62-
49-
38-
28-
17-
14-
6-
3-
GAPDH

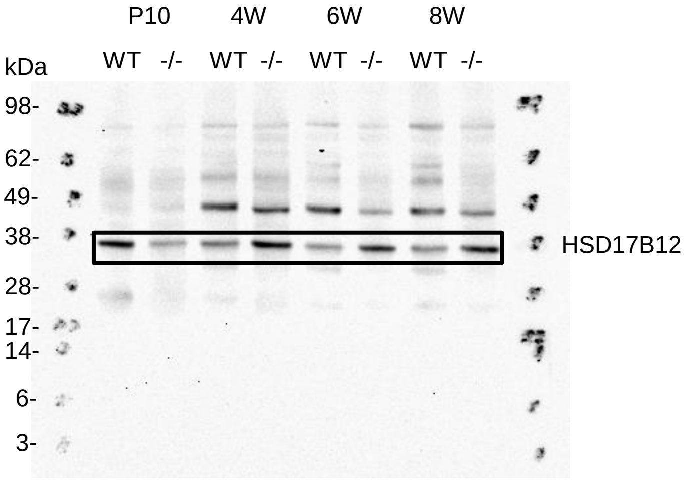
P10 4W 6W 8W
WT -/- WT -/- WT -/- WT -/-
kDa
98-
62-
49-
38-
28-
17-
14-
6-
3-
HSD17B12

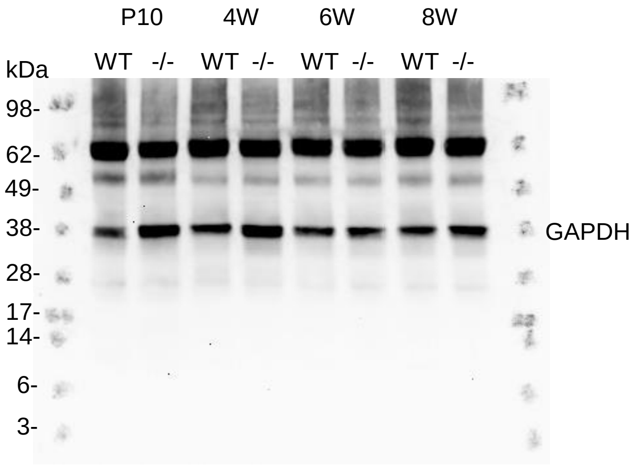
P10 4W 6W 8W
WT -/- WT -/- WT -/- WT -/-
kDa
98-
62-
49-
38-
28-
17-
14-
6-
3-
GAPDH

Fig. S12: Uncropped images of Western Blots Fig. 8b

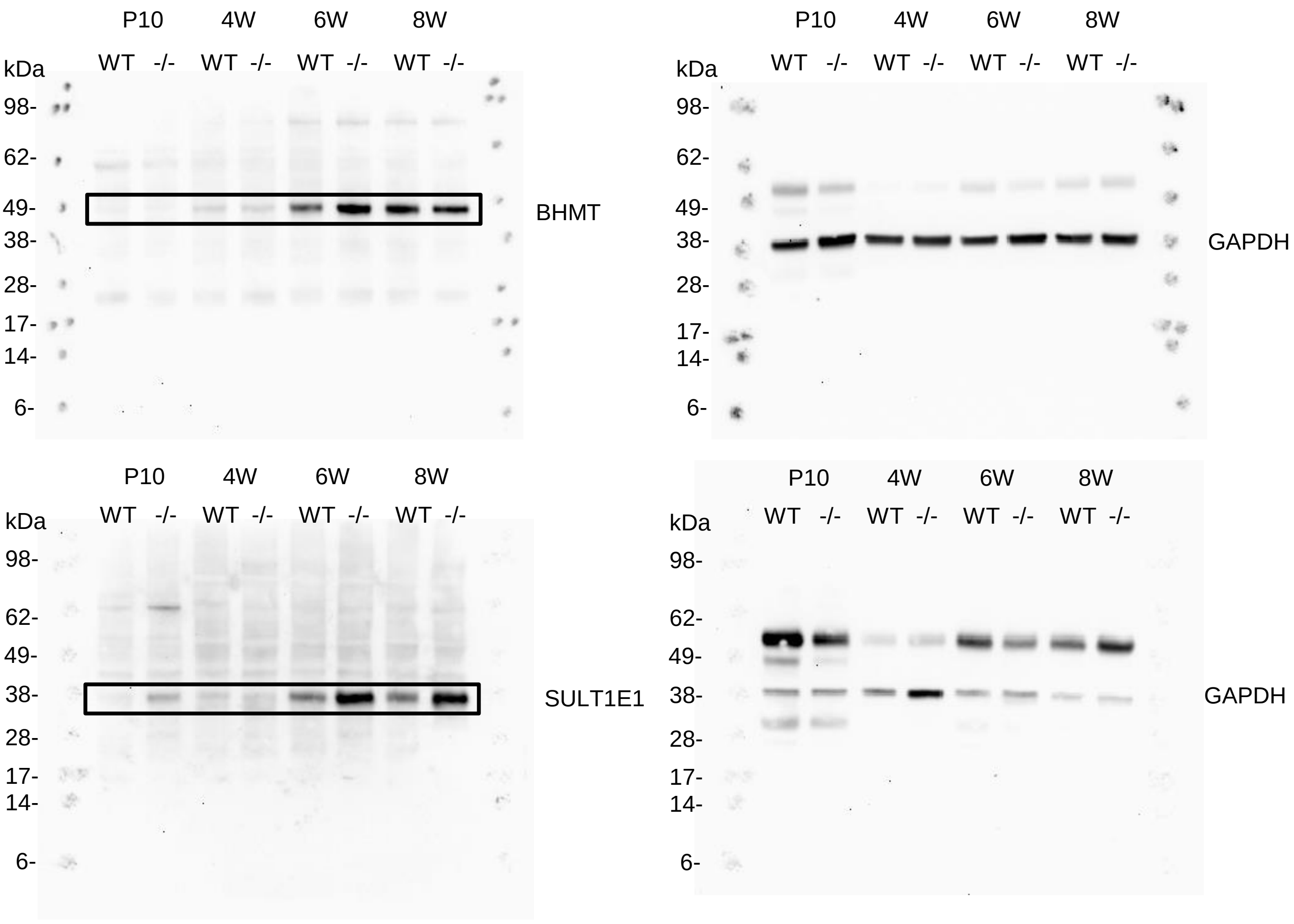

Fig. S13: Uncropped images of Western Blots Fig. 9c

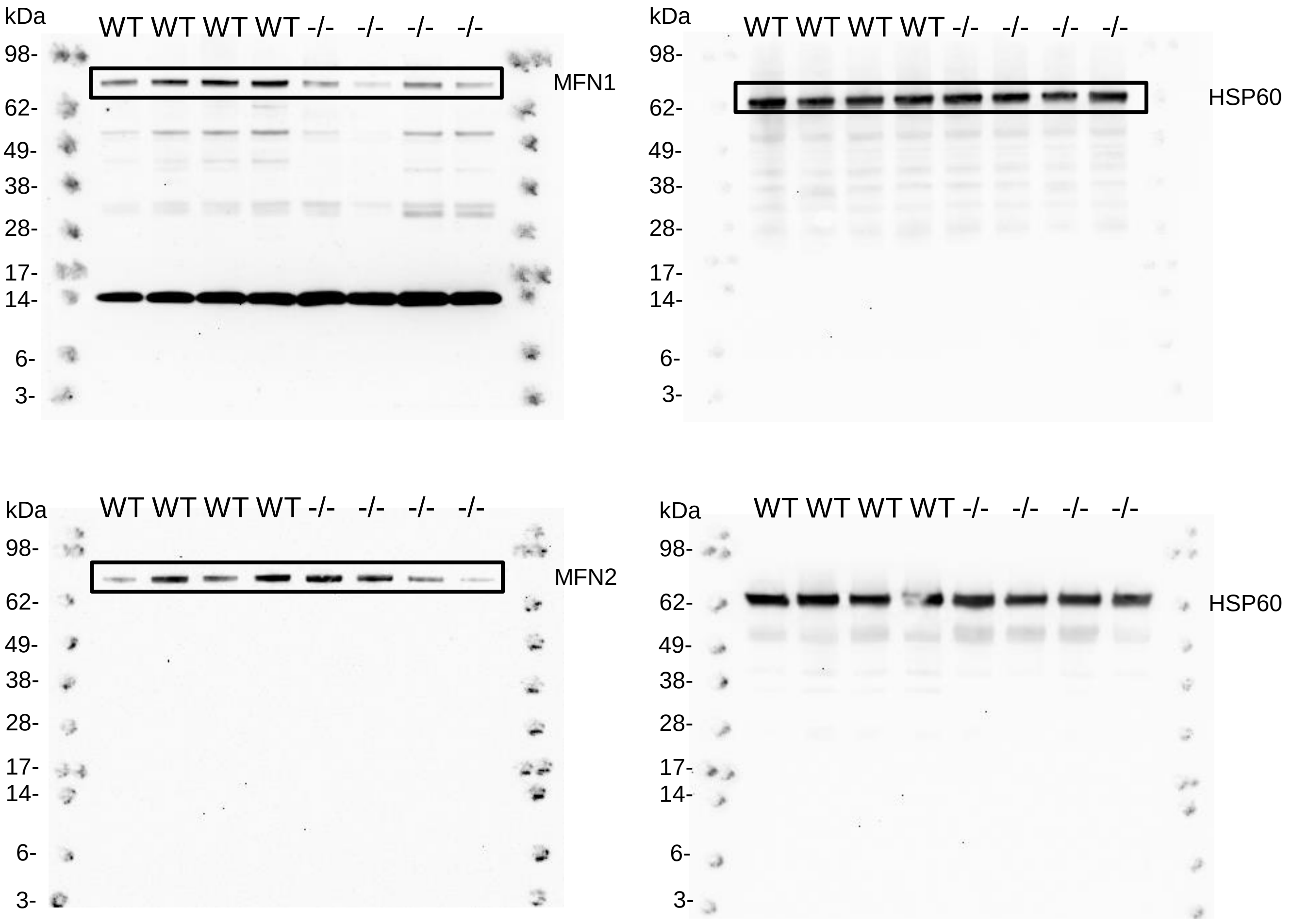

Fig. S14: Uncropped images of Western Blots Fig. S3c

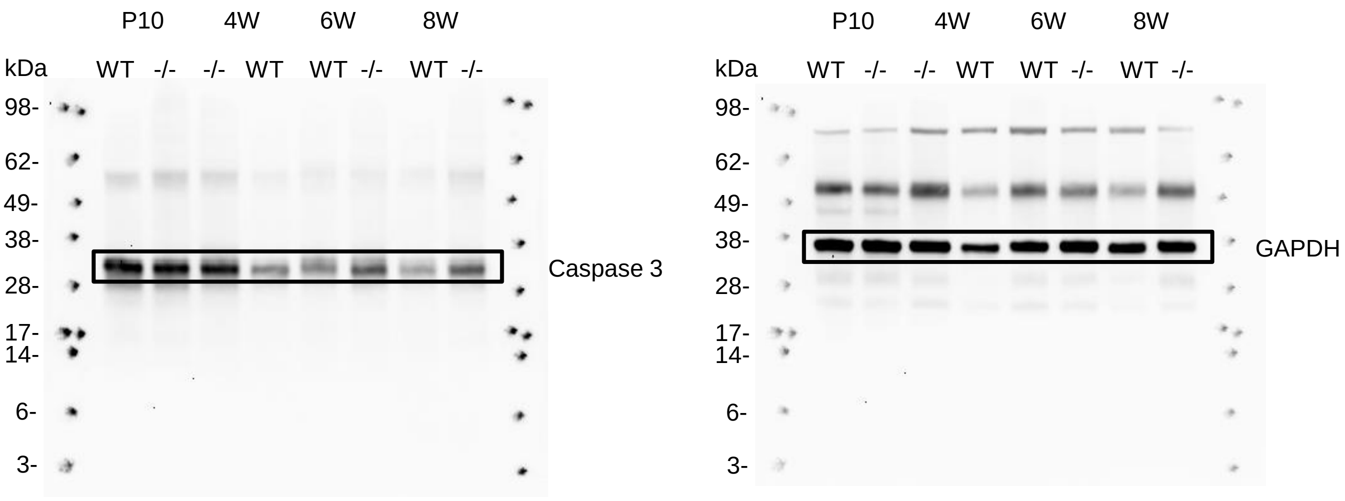

Fig. S15: Uncropped images of Western Blots Fig. S5b

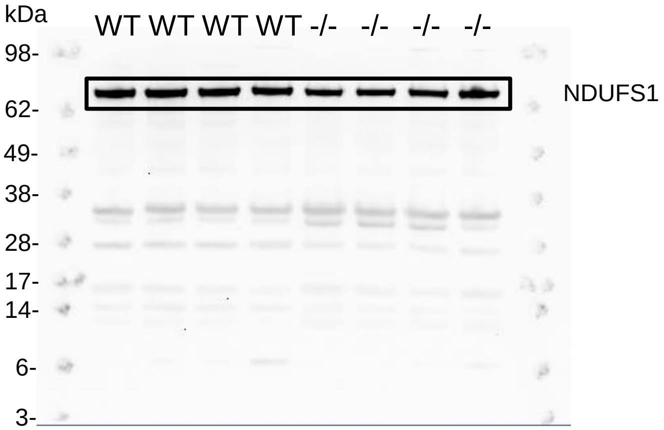

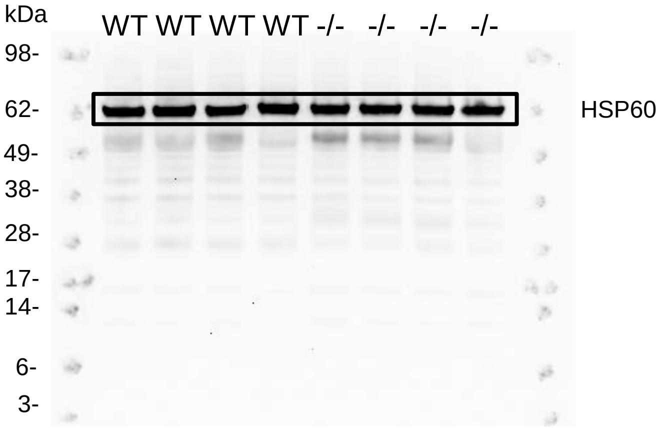

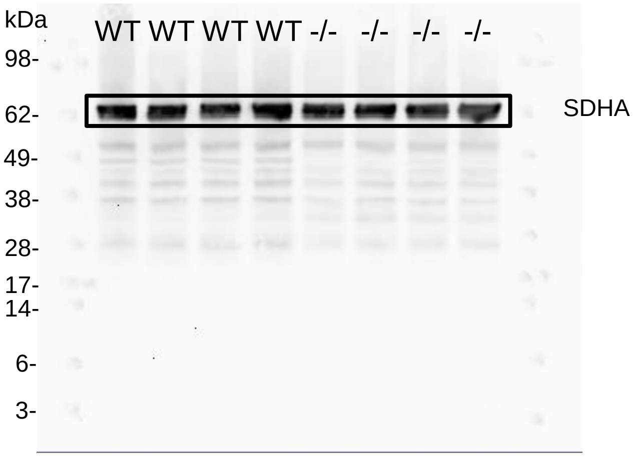

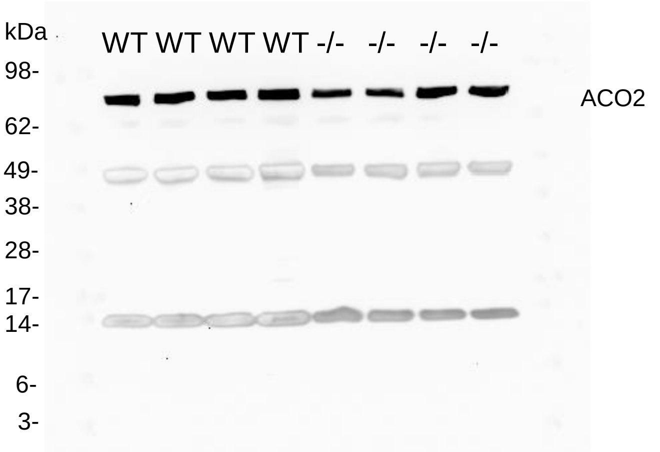

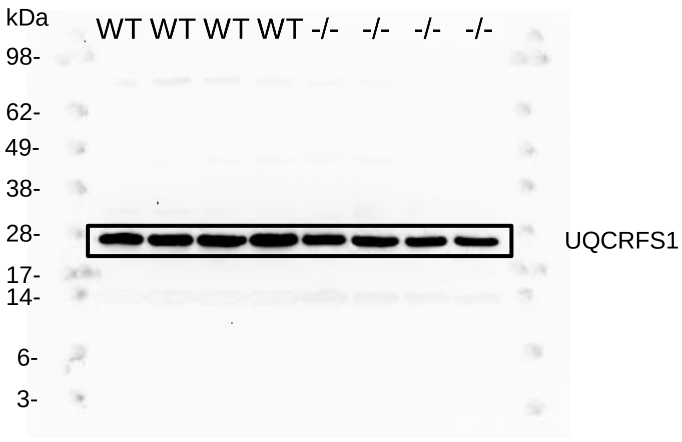

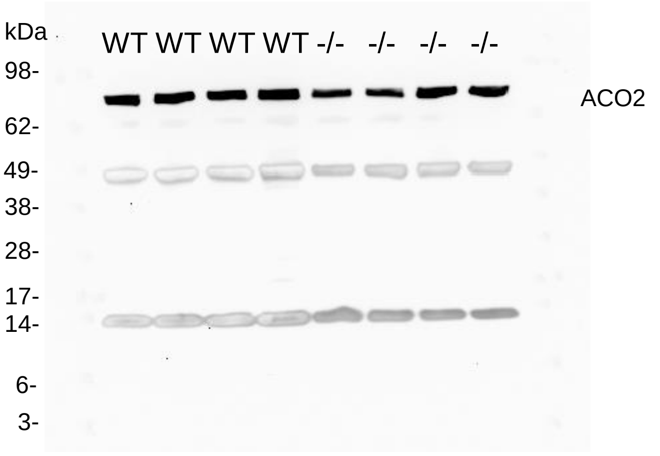

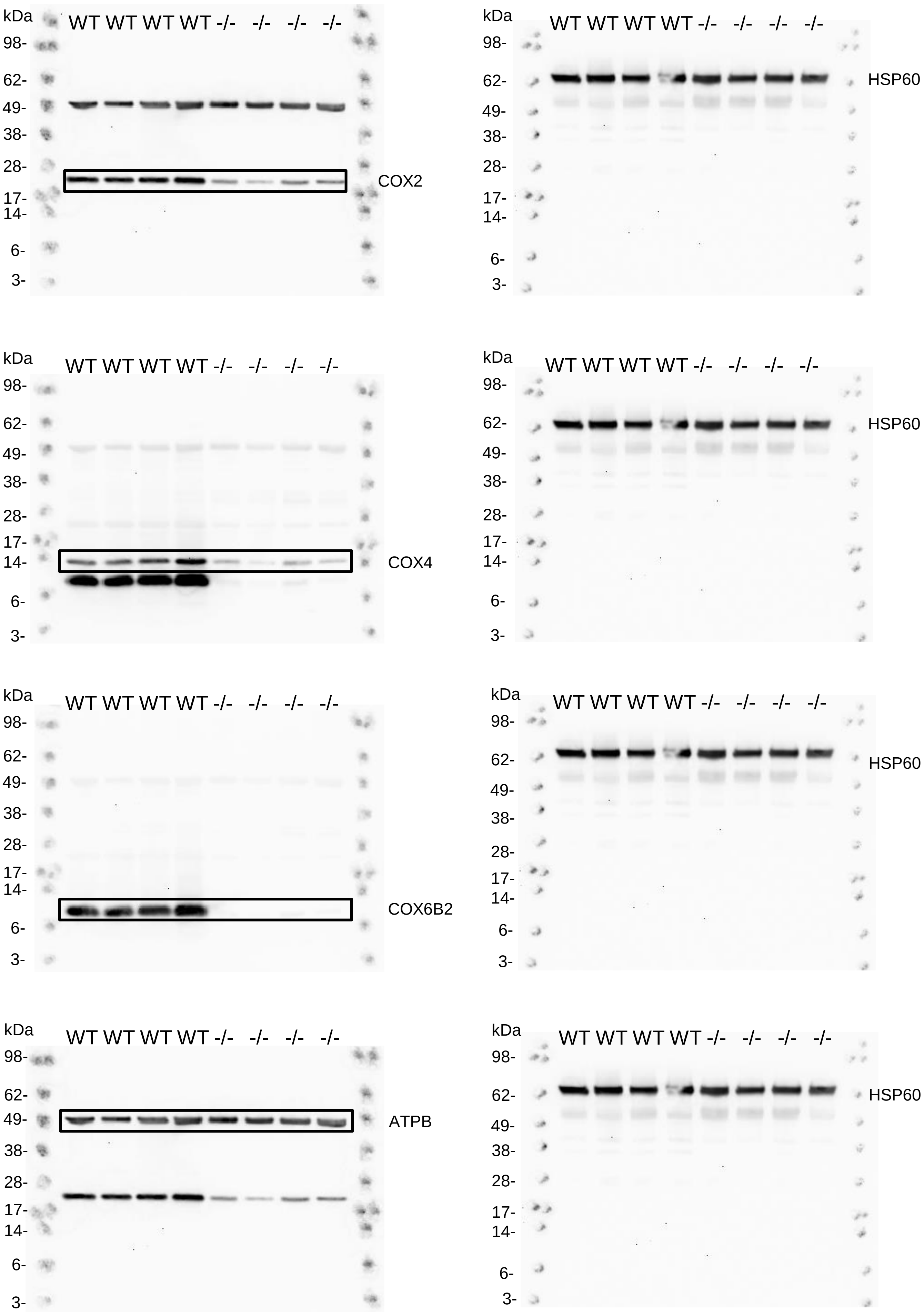
kDa
WT WT WT WT -/- -/- -/- -/-
98- 62- 49- 38- 28- 17- 14- 6- 3-
COX2
HSP60
COX4
COX6B2
ATPB
