# Supplementary material for: The mitochondrial protease PARL is required for spermatogenesis
Source: Commun Biol. 2024 Jan 5;7:44. doi: 10.1038/s42003-023-05703-3 (PMC10770312; doi:10.1038/s42003-023-05703-3)
Supplement: Supplementary file 2 — Description of additional supplementary files [file 42003_2023_5703_MOESM2_ESM.docx]

Description of Additional Supplementary Files

**File name:** Supplementary Data 1

**Description:** Source Data of the graphs in the paper
