# Supplementary material for: The mitochondrial protease PARL is required for spermatogenesis
Source: Commun Biol. 2024 Jan 5;7:44. doi: 10.1038/s42003-023-05703-3 (PMC10770312; doi:10.1038/s42003-023-05703-3)
Supplement: Supplementary file 4 — Reporting Summary - Flat [file 42003_2023_5703_MOESM4_ESM.pdf]

nature portfolio

Corresponding author(s): Sarah Schumacher, Lars Fester

Last updated by author(s): Dec 7, 2023

Reporting Summary

Nature Portfolio wishes to improve the reproducibility of the work that we publish. This form provides structure for consistency and transparency in reporting. For further information on Nature Portfolio policies, see our Editorial Policies and the Editorial Policy Checklist.

Statistics

For all statistical analyses, confirm that the following items are present in the figure legend, table legend, main text, or Methods section.

n/a	Confirmed	
☐	☒	The exact sample size (n) for each experimental group/condition, given as a discrete number and unit of measurement
☐	☒	A statement on whether measurements were taken from distinct samples or whether the same sample was measured repeatedly
☐	☒	The statistical test(s) used AND whether they are one- or two-sided<br>*Only common tests should be described solely by name; describe more complex techniques in the Methods section.*
☒	☐	A description of all covariates tested
☐	☒	A description of any assumptions or corrections, such as tests of normality and adjustment for multiple comparisons
☐	☒	A full description of the statistical parameters including central tendency (e.g. means) or other basic estimates (e.g. regression coefficient) AND variation (e.g. standard deviation) or associated estimates of uncertainty (e.g. confidence intervals)
☐	☒	For null hypothesis testing, the test statistic (e.g. F, t, r) with confidence intervals, effect sizes, degrees of freedom and P value noted<br>*Give P values as exact values whenever suitable.*
☒	☐	For Bayesian analysis, information on the choice of priors and Markov chain Monte Carlo settings
☒	☐	For hierarchical and complex designs, identification of the appropriate level for tests and full reporting of outcomes
☒	☐	Estimates of effect sizes (e.g. Cohen's d, Pearson's r), indicating how they were calculated

Our web collection on statistics for biologists contains articles on many of the points above.

Software and code

Policy information about availability of computer code

Data collection	Nikon EZ-C1 3.91, NIS-Elements AR 3.2, i-control 2.0 (for infinite reader), Quantity One 24.0.0
Data analysis	Graphpad Prism 9.0.0, NIS-Elements AR Analysis 3.2, Nikon EZ-C1 FreeViewer 3.80.860, Microsoft Excel 2010

For manuscripts utilizing custom algorithms or software that are central to the research but not yet described in published literature, software must be made available to editors and reviewers. We strongly encourage code deposition in a community repository (e.g. GitHub). See the Nature Portfolio guidelines for submitting code & software for further information.

Data

Policy information about availability of data

All manuscripts must include a data availability statement. This statement should provide the following information, where applicable:

- Accession codes, unique identifiers, or web links for publicly available datasets
- A description of any restrictions on data availability
- For clinical datasets or third party data, please ensure that the statement adheres to our policy

Data are available from the corresponding authors on reasonable request.

Research involving human participants, their data, or biological material

Policy information about studies with human participants or human data. See also policy information about sex, gender (identity/presentation), and sexual orientation and race, ethnicity and racism.

Reporting on sex and gender	*Use the terms sex (biological attribute) and gender (shaped by social and cultural circumstances) carefully in order to avoid confusing both terms. Indicate if findings apply to only one sex or gender; describe whether sex and gender were considered in study design; whether sex and/or gender was determined based on self-reporting or assigned and methods used. Provide in the source data disaggregated sex and gender data, where this information has been collected, and if consent has been obtained for sharing of individual-level data; provide overall numbers in this Reporting Summary. Please state if this information has not been collected. Report sex- and gender-based analyses where performed, justify reasons for lack of sex- and gender-based analysis.*
Reporting on race, ethnicity, or other socially relevant groupings	*Please specify the socially constructed or socially relevant categorization variable(s) used in your manuscript and explain why they were used. Please note that such variables should not be used as proxies for other socially constructed/relevant variables (for example, race or ethnicity should not be used as a proxy for socioeconomic status). Provide clear definitions of the relevant terms used, how they were provided (by the participants/respondents, the researchers, or third parties), and the method(s) used to classify people into the different categories (e.g. self-report, census or administrative data, social media data, etc.) Please provide details about how you controlled for confounding variables in your analyses.*
Population characteristics	*Describe the covariate-relevant population characteristics of the human research participants (e.g. age, genotypic information, past and current diagnosis and treatment categories). If you filled out the behavioural & social sciences study design questions and have nothing to add here, write "See above."*
Recruitment	*Describe how participants were recruited. Outline any potential self-selection bias or other biases that may be present and how these are likely to impact results.*
Ethics oversight	*Identify the organization(s) that approved the study protocol.*

Note that full information on the approval of the study protocol must also be provided in the manuscript.

Field-specific reporting

Please select the one below that is the best fit for your research. If you are not sure, read the appropriate sections before making your selection.

☒ Life sciences ☐ Behavioural & social sciences ☐ Ecological, evolutionary & environmental sciences

For a reference copy of the document with all sections, see nature.com/documents/nr-reporting-summary-flat.pdf

Life sciences study design

All studies must disclose on these points even when the disclosure is negative.

Sample size	In most experiments n=4 was chosen as a compromise for allowing statistical analysis and the limitted availability of knock-out mice in accordance with the 3R principles.
Data exclusions	No data were excluded from this study.
Replication	At least two technical and biological replicates were performed for all experiments and replication was successful.
Randomization	Animals were allocated to groups based on their genotype.
Blinding	There was no blinding during the experiments. The reported results mostly rely on objectively measured data, so blinding was not necessary.

Reporting for specific materials, systems and methods

We require information from authors about some types of materials, experimental systems and methods used in many studies. Here, indicate whether each material, system or method listed is relevant to your study. If you are not sure if a list item applies to your research, read the appropriate section before selecting a response.

Materials & experimental systems

n/a	Involved in the study
☐	☒ Antibodies
☒	☐ Eukaryotic cell lines
☒	☐ Palaeontology and archaeology
☐	☒ Animals and other organisms
☒	☐ Clinical data
☒	☐ Dual use research of concern
☒	☐ Plants

Methods

n/a	Involved in the study
☒	☐ ChIP-seq
☒	☐ Flow cytometry
☒	☐ MRI-based neuroimaging

Antibodies

Antibodies used

ABP: Abcam, Cat.-No.:ab126617, Lot.-No.: GR3271453-1, Polyclonal Rabbit;
ACO2: Abcam, Cat.-No.: ab129069, lot-No.: GR81376-16, Polyclonal Rabbit;
ACRV1: Proteintech, Cat.-No.: 14040-1-AP, lot-No.: 0004876, Polyclonal Rabbit;
ATPB: Proteintech, Cat.-No.: 17247-1-AP, Lot-No.: 00052097, Polyclonal Rabbit;
BHMT: Novus Bio, Cat No.: NBP1-00161, Lot-No.: P1; Polyclonal Goat;
Caspase3: Cellsignaling, Cat.-No.: 9662, Lot-No.: P42574, Polyclonal Rabbit;
Claudin11: Abcam, Cat.-No.: ab53041, Lot-No.: GR64452-33, Polyclonal Rabbit;
COQ4: Proteintech, Cat.-No.: 16654-1-AP, Lot-No.: 00050107, Polyclonal Rabbit;
COX2 (MTCO2): Proteintech, Cat.-No.: 55070-1-AP, Lot-No.: 00044609, Polyclonal Rabbit;
COX4: Proteintech, Cat.-No.: 11242-1-AP, Lot-No.: 00093375, Polyclonal Rabbit;
COX6B2: Proteintech, Cat.-No.: 11437-1-AP, Lot-No.: 0001974, Polyclonal Rabbit;
CYP11A1: Proteintech, Cat.-No.: 13363-1-AP, Lot-No.: 00046802, Polyclonal Rabbit;
Cyp17A1: Proteintech, Cat.-No.: 14447-1-AP, Lot-No.: -, Polyclonal Rabbit;
DDX4: Abcam, Cat.-No.: ab183840, Lot-No.: 3274948-1, Polyclonal Rabbit;
Diablo(SMAC): Proteintech, Cat.-No.: 10434-1-AP, Lot-No.: 00060963, Polyclonal Rabbit;
GAPDH: HyTest, Cat.-No.: HyTest 5G4, Lot-No.: 16/06-G4-C5, Monoclonal Mouse;
HSD17B3: Biorbyt, Cat.-No.: BYT-ORB5476, Lot-No.: 00041870, Polyclonal Rabbit;
HSD17B12: Invitrogen, Cat.-No.: PA5-69454, Lot-No.: 13706647, Polyclonal Rabbit;
HSP60: Proteintech, Cat.-No.: 66041-1-lg, Lot-No.: 10003124, Monoclonal Mouse;
INHBA: Proteintech, Cat.-No.: 17524-1-AP, Lot-No.: 00089136, Polyclonal Rabbit;
MFN1: Proteintech, Cat.-No.: 13798-1-AP, Lot-No.: 00070973, Polyclonal Rabbit;
MFN2: Proteintech, Cat.-No.: 12186-1-AP, Lot-No.: 00090824, Polyclonal Rabbit;
NDUFS1: Proteintech, Cat.-No.: 12444-1-AP, Lot-No.: 00075263, Polyclonal Rabbit;
PCNA: Abcam, Cat.-No.: ab18197, Lot-No.: GR319234-1, Polyclonal Rabbit;
PGAM5: Abcam, Cat.-No.: ab126534, Lot-No.: GR3182013-21, Polyclonal Rabbit;
SCP1: Novus Bio, Cat.-No.: NB300-229SS, Lot-No.: H-2, Polyclonal Rabbit;
SDHA: Abcam, Cat.-No.: ab14715, Lot-No.: GR3301506-15, Polyclonal Rabbit;
SQRDL: Novus Bio, Cat.-No.: NBP1-84510, Lot-No.: 00033290, Polyclonal Rabbit;
StAR: Proteintech, Cat.-No.: 12225-1-AP, Lot-No.: 000777924, Polyclonal Rabbit;
STARD7: Proteintech, Cat.-No.: 15689-1-AP, Lot-No.: 0006995, Polyclonal Rabbit;
SULT1E1: Proteintech, Cat.-No.: 12522-1-AP, Lot-No.: 00013248, Polyclonal Rabbit;
TCFL5: Proteintech, Cat.-No.: 29404-1-AP, Lot-No.: 00097887, Polyclonal Rabbit;
Transferrin: Proteintech, Cat.-No.: 17435-1-AP, Lot-No.: 00097447, Polyclonal Rabbit;
TTC19: SigmaAldrich, Cat.-No.: HPA052380, Lot-No.: R67698, Polyclonal Rabbit;
UQCRFS1: Proteintech, Cat.-No.: 18443-1-AP, Lot-No.: 0006018, Polyclonal Rabbit;
Vimentin: Abcam, Cat.-No.: ab92547, Lot-No.: GR3186827-13, Polyclonal Rabbit

Validation

Recombinant Anti-SHBG antibody (ABP): tested applications: WB in Human, predicted and cited reactivity in Mouse (Wang Q et al. The anti-androgenic effects of cypermethrin mediated by non-classical testosterone pathway activation of mitogen-activated protein kinase cascade in mouse Sertoli cells. 2019, Ecotoxicol Environ Saf 177:58-65.)
Recombinant Anti-Aconitase 2 antibody (ACO2): tested applications: WB, IHC-P in Human, predicted and cited reactivity in Mouse (Huichalaf et al. In vivo overexpression of frataxin causes toxicity mediated by iron-sulfur cluster deficiency. 2022, Mol Ther MethodsClin Dev.)
ACRV1 Polyclonal Antibody: tested applications: IF, IHC, IP, WB,ELISA in Human cited reactivity in Mouse (Ding et al. TDRD5 binds piRNA precursors and selectively enhances pachytene piRNA processing in mice. 2018, Nat Commun + 4 more);
ATPB Polyclonal Antibody: tested applications: IF, IHC, IP, WB, ELISA; tested reactivity: Human, Mouse, Rat;
BHMT Antibody: tested applications: WB, ELISA, IHC; Reactivity: Human, Mouse, Rat, Canine;
Caspase-3 Antibody applications: WB, IP, IHC; Reactivity: Human, Mouse, Rat, Monkey;
Anti-Oligodendrocyte Specific Protein polyclonal antibody (PCNA): suitable for: ICC, WB; reacts with: Mouse, Rat;
COQ4 Polyclonal Antibody: tested applications: IHC, IP, WB; tested reactivity: Human, Mouse, Rat;
MTCO2 Polyclonal Antibody (COX2): tested applications: FC, IF, IHC, IP, WB; tested reactivity: Human, Mouse, Rat;
COXIV Polyclonal Antibody: tested applications: FC, IF, IHC, IP, WB; tested reactivity: Human, Mouse, Rat;
COX6B2 Polyclonal Antibody: tested applications: IHC, WB; tested reactivity: Human, Mouse, Rat;
CYP11A1 Polyclonal Antibody: tested applications: IHC, WB; tested reactivity: Human, Mouse, Rat;
CYP17A1 Polyclonal Antibody: tested applications: IF, IHC, WB; testes reactivity: Human, Mouse, Rat;
Anti-DDX4 Polyclonal Antibody: suitable for: WB, ICC/IF, IHC-P, IHC-Fr; reacts with: Human, Mouse;
DIABLO Polyclonal Antibody: tested applications: FC, IF, IHC, IP, WB; tested reactivity: Human, Mouse, Rat;
GAPDH antibody: applications: WB, IHC; reacts with: Human, Bovine, Porcine, Goat, Canine, Rabbit, Cat, Rat, Mouse, Fish;

HSD17B3 antibody: tested applications: ELISA, FC, ICC, IF, IHC-Fr, IHC-P, WB; reactivity: Human, Mouse, Rat;
HSD17B12 Polyclonal Antibody: applications: WB; species reactivity: Mouse;
HSP60 Monoclonal antibody: tested applications: WB, IF, IHC, IP,FC; tested reactivity Human, Mouse, Rat
Inhibin beta A-Specific Polyclonal antibody (INHBA): tested applications: WB, IP; tested reactivity Human, Mouse, Rat
MFN1 Polyclonal antibody: tested applications: WB, IHC, IF; tested reactivity Human, Mouse, Rat
MFN2 Polyclonal antibody: tested application WB, IP, IHC; tested reactivity Human, Mouse, Rat
NDUFS1 Polyclonal Antibody: tested applications: IF, IHC, IP, WB; tested reactivity: Human, Mouse, Rat;
Anti-PCNA Polyclonal Antibody: suitable for: WB, ICC/IF, Flow Cyt (Intra), IHC P; reacts with: Mouse, Rat, Human, Common marmoset;
Anti-PGAM5 polyclonal antibody: suitable for: ICC/IF, WB, IHC-P; reacts with: Human, cited with Mouse (Lu W et al. Genetic deficiency of the mitochondrial protein PGAM5 causes a Parkinson's-like movement disorder. Nat Commun 5:4930 (2014) + 1 more);
SCP1 antibody: applications: WB, Simple Western, ChIP, ICC/IF, IHC, IHC-Fr, IHC-P, IP; reactivity: Mouse, Rat, Chicken, Primate;
Anti-SDHA antibody: suitable for: IHC-Fr, Flow Cyt, WB, ICC, IHC-P; reacts with: Mouse, Rat, Cow, Human;
SQRDL antibody: applications: WB, ICC/IF, IHC, IHC-P; reactivity: Human, Mouse, Rat;
STAR Polyclonal Antibody: tested applications: IF, IHC, WB; tested reactivity: Human, Mouse, Rat, Pig;
STARD7 Polyclonal Antibody: tested applications: IHC, WB; tested reactivity: Human, Moues, Rat;
SULT1E1 Polyclonal antibody: tested applications: WB, IP, IHC; tested reactivity Human, Mouse, Rat
TCFL5 Polyclonal Antibody: tested applications: IHC, WB; tested reactivity: Human, Mouse, Rat;
Transferrin Polyclonal antibody: tested applications: WB, IHC, IF; tested reactivity: Human, Mouse, Rat;
TTC19 antibody: techniques: WB, IF, IHC; species reactivity: Human, cited reactivity: Mouse (Spinazzi et al. 2018 PARL deficiency in mouse causes Complex III defects, coenzyme Q depletion, and Leigh-like syndrome. Proc Natl Acad Sci);
UQCRFS1 Polyclonal Antibody: tested applications IHC, IP, WB; tested reactivity: Human, Mouse, Rat;
Recombinant Anti-Vimentin antibody: Suitable for: Flow Cyt (Intra), ICC/IF, WB, IHC-P, mIHC; reacts with: Mouse, Rat, Human, African green monkey

Animals and other research organisms

Policy information about studies involving animals; ARRIVE guidelines recommended for reporting animal research, and Sex and Gender in Research

Laboratory animals	spieces: mouse, strain: Parltm1.1Bdes (+/+, +/- and -/-), age: 10 days, 20 days, 4 weeks, 6 weeks and 8 weeks
Wild animals	The study did not involve wild animals.
Reporting on sex	The study reports mostly results on male animals (testis samples), in some expermiments, reporting on other organ samples than testis, male and female samples were used.
Field-collected samples	The study did not involve field-collected samples.
Ethics oversight	Animal handling was performed in strict accordance to governmental Directive 2010/63/EU of the European Parliament and of the Council of September 22, 2010 further amended by regulation (EU) 2019/1010, institutional animal care regulations and ARRIVE guidelines.

Note that full information on the approval of the study protocol must also be provided in the manuscript.
